# Unravelling genotype-phenotype correlations in Stargardt disease using patient-derived retinal organoids

**DOI:** 10.1038/s41419-025-07420-7

**Published:** 2025-02-19

**Authors:** Avril Watson, Rachel Queen, Luis Ferrández-Peral, Birthe Dorgau, Joseph Collin, Andrew Nelson, Rafiqul Hussain, Jonathan Coxhead, Michael McCorkindale, Robert Atkinson, Darin Zerti, Valeria Chichagova, Ana Conesa, Lyle Armstrong, Frans P. M. Cremers, Majlinda Lako

**Affiliations:** 1 Biosciences Institute, Newcastle University, Newcastle upon Tyne, UK; 2 Newcells Biotech Ltd., Newcastle upon Tyne, UK; 3 Institute for Integrative Systems Biology, University of Valencia, Valencia, Spain; 4 NU-OMICs, Northumbria University, Newcastle Upon Tyne, UK; 5 Department of Biotechnological and Applied Clinical Sciences, Università degli Studi dell’Aquila, L’Aquila, Italy; 6 Department of Human Genetics, Radboud University Medical Center, Nijmegen, the Netherlands

**Keywords:** Stem cells, Pluripotent stem cells

## Abstract

Stargardt disease is an inherited retinopathy affecting approximately 1:8000 individuals. It is characterised by biallelic variants in *ABCA4* which encodes a vital protein for the recycling of retinaldehydes in the retina. Despite its prevalence and impact, there are currently no treatments available for this condition. Furthermore, 35% of STGD1 cases remain genetically unsolved. To investigate the cellular and molecular characteristics associated with STGD1, we generated iPSCs from two monoallelic unresolved (PT1 & PT2), late-onset STGD1 cases with the heterozygous complex allele - c.[5461-10 T > C;5603 A > T]. Both patient iPSCs and those from a biallelic affected control (AC) carrying -c.4892 T > C and c.4539+2001G > A, were differentiated to retinal organoids, which developed all key retinal neurons and photoreceptors with outer segments positive for ABCA4 expression. We observed patient-specific disruption to lamination with OPN1MW/LW^+^ cone photoreceptor retention in the retinal organoid centre during differentiation. Photoreceptor retention was more severe in the AC case affecting both cones and rods, suggesting a genotype/phenotype correlation. scRNA-Seq suggests retention may be due to the induction of stress-related pathways in photoreceptors. Whole genome sequencing successfully identified the missing alleles in both cases; PT1 reported c.-5603A > T in homozygous state and PT2 uncovered a rare hypomorph - c.-4685T > C. Furthermore, retinal organoids were able to recapitulate the retina-specific splicing defect in PT1 as shown by long-read RNA-seq data. Collectively, these results highlight the suitability of retinal organoids in STGD1 modelling. Their ability to display genotype-phenotype correlations enhances their utility as a platform for therapeutic development.

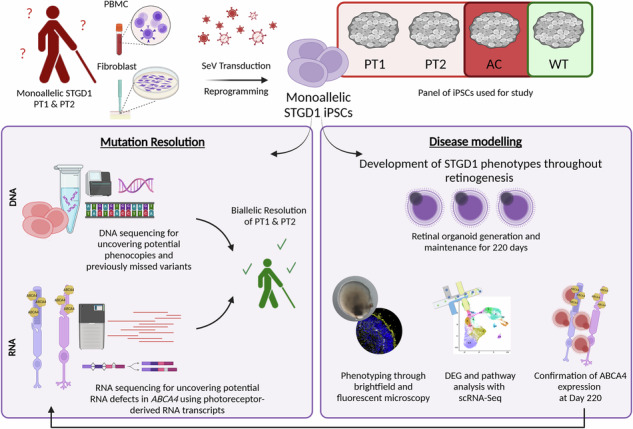

## Introduction

Stargardt disease (STGD1; OMIM #248200) is the leading cause of inherited macular degeneration among working-aged individuals. Whilst a global incidence of 1:8000 affected is generally accepted in published studies [1], more recent figures report the incidence as 1:19,000-20,000 [2]. “Classic” STGD1 is described as a juvenile maculopathy that becomes apparent within the first two decades of life. As a macular condition, bilateral central vision is mostly affected. Initial symptoms include decreased visual acuity, defects with chromatic vision, and photophobia, which cannot be aided by corrective eyewear [3]. Despite the continued progression of central vision loss over time, peripheral vision tends to be completely spared in STGD1.

As a monogenic disease, STGD1 is caused solely by biallelic variants in ***A****TP-****b****inding* ***c****assette Subfamily* ***A*** *Member* ***4*** (*ABCA4*), a gene expressed exclusively in the retina – primarily in photoreceptor outer segments (POS) of both rod and cones [4], but also in the retinal pigment epithelium (RPE) [5]. *ABCA4* contains 50 exons in a 128-kb region gene which boasts an expansive and ever-growing list of genetic variation. Over 1000 pathogenic/likely-pathogenic variants have thus far been reported in both coding and non-coding regions of the gene. In addition to this, there is large degree of polymorphism in the gene with an overall carrier incidence of 5% in the general population [6–10].

ABCA4 is an important mediator in the recycling of visual pigments following phototransduction. It functions as a ‘flippase’ in the POS to transport retinoid conjugates - N-retinylidene-phosphatidylethanolamine (NRPE), from the inner lumen to the cytoplasmic space, where all-*trans*-retinal can be further metabolised and regenerated to 11-*cis-*retinal for further rounds of phototransduction [11, 12]. In STGD1, this process is compromised due to reduced function of ABCA4 flippase activity, resulting in a build-up of NRPE and formation of A2-phosphatidylethanolamine (A2-PE) inside the POS disc lumen. Diurnal phagocytosis of POS by adjacent RPE cells results in the entry of A2-PE molecules into the phagolysosome, where the acidic environment encourages the formation of A2E, a pyridinium bisretinoid and known component of lipofuscin [13]. Lipofuscin is toxic to cells and in the context of STGD1 is classically understood to be the cause of RPE cell degeneration and secondary photoreceptor cell loss in the fovea.

At present, obtaining a genetic diagnosis for STGD1 is relatively straight forward in identifying biallelic pathogenic variants in the coding regions of *ABCA4*. With the use of cost-effective target panel capture systems with next-generation sequencing (NGS) [14] and the development of single molecule molecular inversion probes for whole gene sequencing of *ABCA4* [15], the solve rate has stabilised for STGD1 diagnosis at ~95%, if including monoallelic cases with strong phenotypic indicators of STGD1 [16]. However, ~15–20% of all STGD1 cases remain unsolved with just one or no pathogenic *ABCA4* variants identified, despite a strong clinical indication of the disease. It was originally hypothesised that late-onset STGD1 cases were intrinsically monoallelic, however this was disproven with the realisation of the common allele c.5603 A > T functioning as a hypomorphic variant, only penetrant when in trans with a null allele [17]. Another source of missing variation in these cases proved to be in the non-coding regions of *ABCA4* resulting in pathogenic RNA defects [18, 19]. Could the remaining 5% of unresolved cases also harbour hypomorphic alleles or RNA defects pertinent in ABCA4-pathology?

To answer this question, we generated induced pluripotent stem cells (iPSCs) from two monoallelic late-onset STGD1 cases and differentiated them into retinal organoids (ROs) alongside affected and unaffected control iPSCs. We phenotyped these ROs over 220 days using immunocytochemistry and assessed their expression levels of *ABCA4* at the transcript and protein levels at the final timepoint to validate this model for studying STGD1. For the first time, we show that STGD1 ROs exhibit a phenotypic defect characterised by photoreceptor mislocalisation, which correlates in intensity with disease severity. Our single-cell RNA sequencing (scRNA-Seq) data demonstrate that STGD1 ROs display gene dysregulation in stress-response and phototransduction pathways, indicating that the model can recapitulate STGD1 phenotypes in vitro*.* Most importantly, we identified the missing alleles in two STGD1 patients using whole genome sequencing (WGS) and long-read RNA sequencing (LRS) technologies.

## Materials & Methods

### Human subjects

All participants in this research study were identified and enlisted by collaborators in the Marie Skłodowska-Curie Innovative Training Network – StarT (Grant no: 813490). Patient 1 (PT1) and Patient 2 (PT2) were enlisted as monoallelic STGD1 cases, both with the same resolved complex allele in *ABCA4*; c.[5461-10 T > C,5603 A > T] p.[Thr1821Valfs*13,Thr1821Aspfs*6;(Asn1868Ile)] and a clinical indication of late-onset STGD1. The affected control (AC) carries two *ABCA4* variants; Allele 1: c.4539+2001G > A, p.[Arg1514Leufs*36,=] and Allele 2: c.4892 T > C, p.(Leu1631Pro) with a classic clinical STGD1 presentation. WT2 & WT3 iPSCs were derived and thoroughly characterised by our research group [20, 21]. All samples were collected in accordance with the tenets of the Declaration of Helsinki and written informed consent was obtained for all patients participating in the study.

### iPSC generation for PT1 & PT2

PT1, AC, and WT2 & 3 samples were provided to us as iPSCs. However, due to culturing difficulties with spontaneous differentiation and inaccessibility to primary material during the Covid19 pandemic period, PT1 was subjected to additional iPSC reprogramming alongside PT2. Mesenchymal-like cells were derived from PT1 iPSCs from culture with Dulbecco’s Modified Eagle Medium:Nutrient Mixture F-12 (DMEM/F-12, Life Technologies, 11330032) with 20% foetal bovine serum (FBS, Life Technologies, 10270106) which we treated as somatic cells for the purpose of reprogramming. PT2 iPSCs were derived from primary peripheral blood mononuclear cells (PBMCs). Both samples were reprogrammed following the Cytotune™-iPS 2.0 Reprogramming Kit (Life Technologies, A16517) to manufacturer specifications specifically for fibroblasts and PBMCs. iPSC colonies were established using the feeder-dependant strategy on mitotically inactivated primary mouse embryonic fibroblasts (iMEFs) (Sigma Aldrich, PMEF-CF) and later adapted to feeder-free conditions. During the transitory phase of feeder to feeder-free conditions, iPSC medium was supplemented with iMEF-conditioned medium at a ratio of 1:1 to ensure continued growth factor supplementation on feeder plates.

### iPSC culture

iPSCs were cultured on 6-well plates coated with Matrigel™ GFR (Corning, 354230) at a concentration of 83 µg/ml per well. iPSCs were maintained in mTeSR™1 (StemCell Technologies, 05850) media supplemented with 1% penicillin/streptomycin (Gibco, 15140) in a humidified incubator at 37°C, 5% CO_2_. Cells were fed daily, any spontaneous differentiation was mechanically removed. Upon reaching 80% confluency, cells were passaged using Versene (EDTA 0.02%) (Lonza, B17-771E) solution at 37 °C for 3-4 minutes and split at 1:3/6 ratio to freshly-coated Matrigel plates. iPSCs were cryopreserved using 90% FBS, 10% DMSO (Sigma Aldrich, D2650) and 10 µM ROCK inhibitor (Chemdea, CD0141).

### iPSC characterisation

Successfully reprogrammed clones of PT1 and PT2 were assayed for clearance of Sendai Viral (SeV) vectors at passage 12 via RT-PCR with pre-defined SeV transgene sequences included in the CytoTune 2.0 manual. Polymerase chain reaction (PCR) products of samples and positive control were separated on 2% agarose gel to ensure specific bands were achieved in positive control but absent in PT1 and PT2 clones. Successfully reprogrammed clones of PT1 and PT2 were externally characterised using ThermoFisher PluriTest and KaryoStat services. PluriTest compares the transcriptomic profile of provided samples against a reference dataset of pluripotent and non-pluripotent cells/tissues to yield a PluriCor and Novelty score to confirm pluripotency in the sample. Karyostat is a microarray that looks for copy number variants (CNV) and single nucleotide polymorphisms (SNP) across the genome in the provided samples enabling the detection of aneuploidies, submicroscopic aberrations, and mosaic events to detect any chromosomal aberrations. iPSC lines were frequently tested for mycoplasma contamination throughout the culture.

### RO differentiation

Two methods of differentiation were used for generating STGD1 ROs from all iPSC lines (PT1, PT2, WT2, WT3 and AC). These protocols are the Bone Morphogenetic Protein 4 (BMP4)-activated method [22] and the Insulin Growth Factor 1 (IGF1)-dependent refined method [23] to favour the M/L-opsin fate of cone photoreceptors, which are enriched in native foveal tissue. Media compositions can be found in the respective publications.

### RO processing

#### Fixation and sectioning

ROs were collected and washed in 1x phosphate buffered solution (PBS) before fixing in 4% paraformaldehyde for 20 minutes at 4 °C. ROs were dehydrated in a sucrose gradient from 6.25% to 30% overnight. The next day, ROs were embedded in moulds (Tebu-Bio, UK 18985–1) using optimum cutting temperature medium (CellPath, KMA-0100-00A) with minimal physical manipulation to prevent POS damage. Samples were then sectioned using a cryostat (Leica, CM1860) at 7–10 μm thickness and placed on Epredia™ SuperFrost Plus™ Adhesion slides (ThermoScientific, 10149870). Sections were stored at -20 °C until immunocytochemical staining.

#### Immunocytochemistry

Sections were assessed under dissection microscope to ensure sufficient structures were present. ROs were then separated using the ImmEdge Pen (VectorLabs, H-4000) and subsequently airdried for 20 minutes post-thaw. The sections were rehydrated with 3 × 5-minute PBS washes. Sections were blocked using blocking serum consisting of 10% normal goat serum and 0.3% Triton-X-100 in PBS for 1 hour at room temperature (RT). Primary antibodies (Table S1) were diluted in PBS with 10% normal goat serum and applied to the slides overnight at 4 °C. The following day, sections were washed using 3 × 10-minute PBS washes. Secondary antibodies in PBS were applied to the slides and incubated for 2 hours at RT. Sections were washed again with PBS and counterstained with Hoechst nuclear stain (Sigma, B2261) for 10 minutes. Slides were mounted using Vectashield (VectorLabs, H-1000).

#### Quantification of Immunocytochemistry

Quantification of positively stained cells was achieved using Zen® Blue Software (Zeiss, Germany) and MATLAB® (MathWorks®, MA) as described in Dorgau, Felemban [24]. Briefly, this script facilitates the cropping of neuroretinal regions from the RO images and the subsequent identification of individual cell types using Hysteresis thresholding techniques to filter background noise. This facilitates the identification of each cell type within blue, green, and red fluorescent channels yielding information regarding cell size, average intensity values and length. Following this, the total size, and the percentage of the positive cells in the red/green channel (retinal markers) that colocalise with cells of the blue channel (Hoechst nuclear marker) are exported to excel and used for assessing the composition of ROs and performing statistical analysis. A minimum of 5 images of ROs across 3 biological repeats in each condition at each time point were used for quantification analysis. Groups compared demonstrated similar variability and error bars were tight. A minimum of 5 unique images gives us the statistical power required to see an effect. For our final timepoint, quantification only utilised ROs that showed a bright-phase neuroepithelium and a brush border, excluding ROs that failed to reach this milestone. ABCA4 and CRALBP could not be quantified using this method, consequently, they were not analysed in this manner.

#### Protein Isolation

16 ROs were collected per iPSC sample at Day 220 of differentiation to assess the expression of ABCA4 protein. Cell lysates were prepared from pelleted ROs using ice-cold RIPA lysis buffer (Millipore, 20188) containing an EDTA-free protease inhibitor cocktail (Sigma Aldrich, 11873580001). Samples were incubated with the lysis buffer for 30 minutes on ice with frequent pipetting at 10-minute intervals to assist in the dissociation and lysis of the cells. Completely lysed samples were centrifuged at 1000 x *g* for 5 minutes at 4 °C. Supernatants were collected and protein concentration quantified using the colorimetric Pierce BSA Protein Assay following manufacturer’s specifications.

#### Western blotting

30 μg of protein from each RO sample were mixed with Novex Tris-Glycine SDS Sample Buffer (Thermofisher, LC2676) and NuPAGE Sample Reducing Agent (Thermofisher, NP0004), and subsequently incubated at 85°C before loading onto Novex™ Tris-Glycine Mini Protein Gels, 4–12%, 1.0 mm, WedgeWell™ format (Thermofisher, XP04125). Samples were separated in Novex Tris-Glycine SDS Running Buffer (Thermofisher, LC2675) alongside PageRuler Plus Prestained Protein Ladder (Thermofisher, 26619). Due to the large size of ABCA4 protein (~250 kDa), the gel rig was run at 90 V for 20 minutes, then increased to 120 V for up to 2 hours. Proteins were transferred to the PVDF membrane via the wet transfer method. The membrane was activated in 10% methanol and the sandwich prepared in the order: 2 sponges, filter paper, gel, membrane, filter paper and 2 sponges. Transfer of samples was conducted at 30 V for 90 minutes in Novex Tris-Glycine Transfer Buffer (Thermofisher, LC3675).

Primary antibodies were incubated with the membrane on a roller overnight at 4 °C (Table S1). The following day, the membrane was incubated with secondary antibodies for 1 hour at RT. The signal was processed using SuperSignal™ West Pico PLUS Chemiluminescent Substrate (Thermofisher, 34580), visualised and analysed by Amersham Imager 600 (GE, USA) with ACTB as a loading control.

### Single Cell RNA sequencing

#### Dissociation of ROs to single cells and sequencing library preparation

A minimum 25 ROs per sample were harvested at Day 200 of differentiation and enzymatically dissociated to single cells using the Neurosphere Dissociation Kit (P) (Miltenyi Biotech, 130-095-943) following the manufacturer’s protocol. Cell capture and sequencing libraries were generated using the Chromium Single Cell 3’ Library and Gel Bead Kit v3.1 (10x Genomics, PN-1000121).

#### Data processing and quality control

Once libraries were generated for each sample, they were sequenced by the Genomics Core Facility at Newcastle University up to 50,000 reads per cell on the NovaSeq 6000 (Illumina). CellRanger mkfastq v3.1 was used to de-multiplex resulting BCL files into FASTQ files. Samples were then aligned to the human reference genome GRCh38 for annotation and subsequent generation of gene expression matrices for each sample. Quality control was carried out in R Studio (Posit) on the annotated reads with thresholds set to remove reads with ≤ 1000 reads or ≤ 500 genes. Cells with mitochondrial reads ≥ 10% were also removed from the dataset. Doublets were identified and removed using DoubletFinder v2.0.3 [25].

For each sample, individual normalisation was performed using Seurat (v3.1.3), an R toolkit designed for single-cell genomics analysis. Subsequently, the data were subjected to dimension reduction through PCA using the top 2000 highly variable genes. To alleviate batch effects within the datasets, we integrated the first 30 principal components of each sample using Harmony v0.1.1, resulting in the creation of a unified integrated dataset. Visualisation of the data involved the utilisation of Uniform Manifold Approximation and Projection (UMAP), which was based on the initial 10 batch-corrected coordinates and the clusters identified by Seurat. Differentially expressed markers between each cluster were identified using the Seurat FindMarkers function with the method Wilcoxon test. These genes were used to group cell types into population clusters on the UMAP at a resolution of 0.5.

RO differentially expressed genes between PT2, AC and WT2 + 3 ROs were subjected to the Ingenuity Pathway Analysis (IPA) Software (Qiagen) to evaluate the functional aspects related to disease and the canonical pathways involved in STGD1.

### Statistical analyses

Statistical analyses on quantified fluorescence data from all ROs were conducted via GraphPad Prism v10 (GraphPad Software, LLC). One-way analysis of variance (ANOVA) (Šídák’s multiple comparisons test) was primarily used to compare mean cell percentages ± standard error mean (SEM) values of each retinal marker within distinct protocol subsets. With this statistical test, PT1 ROs were compared with AC and WT2 ROs in the IGF1-dependent method, and PT2 ROs were compared with WT2 ROs in the BMP4-activated method. The data was normally distributed and one-way ANOVA was also used in the same manner for assessing the relative expression of ABCA4 in Western blotting experiments across all samples at Day 220 of differentiation.

Statistics for scRNA-Seq experiments were conducted on R-Studio. The *p*-values associated with the plots in this dataset are from the Fisher’s Exact test which was used to measure the differences in abundances between different phases of cell cycle and abundance of apoptotic transcripts within photoreceptors across samples. Values of *p* ≤ 0.05 were considered statistically significant (* = *p*-val ≤ 0.05, ** = *p*-val ≤ 0.01, *** = *p*-val ≤ 0.001, **** = *p*-val ≤ 0.0001).

### WGS

#### DNA Isolation

DNA was isolated from PT1, PT2, WT2 and AC iPSCs for the purpose of WGS and variant validation thereafter. iPSCs were pelleted and DNA was isolated using the QIAamp DNA Mini Kit (Qiagen, 56304) according to manufacturer specifications with extra optional steps to increase overall yield. Samples were eluted into 200 μl of nuclease-free water. DNA concentrations were quantified by NanoDrop, and samples were stored at -20 °C until later use.

#### WGS of Samples

WGS on PT1 and PT2 iPSC-DNA was carried out by the Genomics Core Facility at Newcastle University. Samples were sequenced on the NovaSeq 6000 Platform using a standardised pipeline. Raw data was then processed by the Bioinformatic Support Unit at Newcastle University. FASTQ files resulting from the sequenced samples were subjected to quality control measures including the removal of low-quality reads and adapter contamination using Cutadapt tool. Duplicates were marked and removed using Picard. Samples were aligned to GRCh38 human genome and annotated for variant calling where single nucleotide variants (SNVs), and small insertions/deletions (indels) were identified using the Genome Analysis Toolkit (Broad Institute). Called variants were filtered on resulting variant call format (VCF) files using a homemade macular disease gene panel. Using Excel, variant prioritisation was carried out based on pathogenicity scores (CADD, SIFT, PolyPhen-2, REVEL) and minor allele frequencies (MAF).

#### Validation of candidate variants from WGS

Variants identified in the VCF files from WGS were validated using Sanger sequencing. Variant-specific primers were designed and desired products from PT1 and PT2 were amplified by PCR. Primers were generated using sequences obtained approximately 250 bp upstream and downstream of the variant of interest (Table S2). These sequences were captured using the Integrated Genome Browser (IGV) genome browser and input into Primer 3 software (https://primer3.ut.ee/) to generate primers yielding products no more than 500 bp in size. In-silico PCR tool (https://genome.ucsc.edu/cgi-bin/hgPcr) was used to ensure no unspecific binding of the primer and only the product of interest was amplified. DNA primers were synthesised by Sigma Aldrich and sent resuspended at 100 μM concentration in nuclease free water. PCR products were visualised via gel electrophoresis and imaged as described previously. Remaining PCR products were purified to remove residual primers using QIAquick PCR Purification Kit (Qiagen, 28104) and sent for Sanger sequencing. The sequence obtained from the Sanger traces was aligned with the reference DNA product using Clustal Omega (https://www.ebi.ac.uk/Tools/msa/clustalo/) to quickly detect mismatches and confirm the variant.

### Long-read RNA sequencing

#### Sample preparation

RNA was harvested from PT1, PT2 and WT2 ROs at Day 220 to ensure the full development of photoreceptor cells producing mature *ABCA4* transcripts for LRS. ROs were cultured with the nonsense-mediated decay inhibitor Puromycin (Sigma Aldrich, P7255) for 7 hours prior to harvest. RNA-integrity number (RIN) was >9 for both samples. RNA isolation was conducted using ReliaPrep™ RNA Miniprep Kit (Promega, Z6112) following manufacturer’s specifications.

#### Sequencing of samples with PacBio LRS

LRS on the RNA samples prepared was carried out by the NU-OMICs facility using the PacBio Sequel sequencer (Pacific Biosciences). RNA sequencing was carried out following the IsoSeq protocol to enable full-length transcriptome sequencing. Briefly, RNA was converted to cDNA and unique molecular barcodes (known as PacBio SMRTbell adaptors) were added to individual cDNA molecules to circularise cDNAs to encourage increased read depth via circularised consensus sequencing. The barcoded library pool was then subjected to real-time SMRT sequencing which produces long read lengths which can span entire transcripts, enabling its use in full-length transcriptome analysis, and in the identification of novel isoforms and alternative splicing events [26].

#### Analysis of raw data from LRS

To calculate percent spliced-in (PSI) for *ABCA4* exons 39 and 40, we first aligned the Iso-Seq HiFi reads from the two patients and the control against hg19 assembly using minimap2 (v. 2.17-r941) with the following mapping parameters: -ax splice:hq -uf --MD -t 12 [27]. SAM files were converted into BAM and then indexed using Samtools [28]. Spliced reads mapping to *ABCA4* were quantified using ggsashimi [29] discarding any splice junctions with only one supportive read. PSI for exon skipping events was calculated considering exon inclusion and exclusion reads as in Saraiva-Agostinho and Barbosa-Morais [30].

Transcript models were generated from high fidelity (HiFi) reads following the official PacBio pipeline. Quality control of generated transcript models in the three samples was performed using SQANTI3 [31, 32] which was also used to predict the open reading frames (ORFs) encoded in the RNA transcripts. The comparison between ABCA4 canonical protein (encoded in PB.390.3 transcript model from PT1) versus the truncated protein (encoded in PB.390.4 transcript model from PT1, which skips exon 39) was represented using tappAS [33]. To include annotated protein features in the representation (e.g., protein domains), transcript coordinates were first projected from hg19 to hg38 using UCSC LiftOver with default settings [34] and then IsoAnnotLite was used to annotate protein features in the predicted ORFs based on Ensembl v86 (hg38).

The three-dimensional representation of ABCA4 (P78363 from UniProt, Fig. S6B) was generated with iCn3D software [35]. 1821-2273 amino acid residues were highlighted to indicate the protein truncation resulting from exon 39 skipping. This protein region was selected based on a pairwise alignment [36] between the predicted coding sequences from PB.390.3 and PB.390.4 transcript models (PT1).

## Results

### STGD1 iPSCs produce ROs with photoreceptor cells expressing ABCA4

STGD1 PT1 and PT2 iPSCs were derived using a non-integrative RNA-based Sendai virus system (Fig. S1A). Several clones from each STGD1 patient were tested for clearance of transgenes (Fig. S1B, C), maintenance of pluripotency, and genome stability (Fig. S1D&E). Except for the clones of STGD1 PT1, which displayed an increased copy number in the Chr 20q11.21 region and loss of the 20p11.1 region (Fig. S1E), all other clones showed the expected copy number in loci most susceptible to changes in pluripotent stem cells.

Both STGD1 patient iPSCs, AC, and WT iPSC lines were differentiated into ROs using two routinely employed protocols in our research group: the BMP4-activated [22], and the IGF1-dependent method [23]. Due to differences in the propensity of individual iPSC lines to differentiate using a single protocol, we chose to use both methods to generate photoreceptors capable of ABCA4 expression. Despite variations in the initial stages of differentiation, ROs generally follow the same transcriptional differentiation trajectory, leading to retinogenesis over the 220-day period, resulting in remarkable similarity by the end point. To minimise the effects of this confounding factor in interpreting our results, we used WT2 as a control, as it’s known to differentiate well with both protocols. This allowed us to control for protocol-specific phenotypes and accurately deduce true STGD1 phenotypes.

**Fig. 1:**
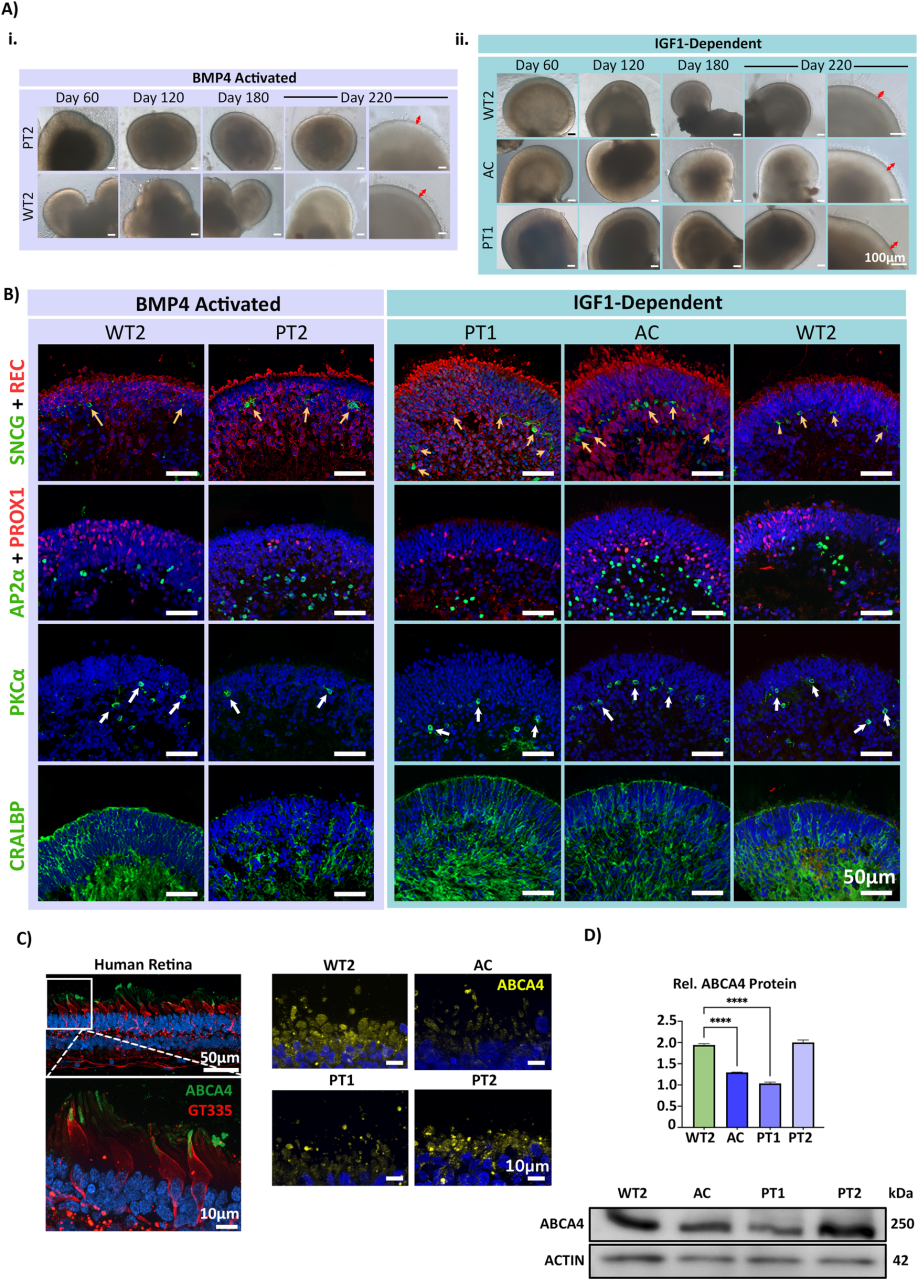
STGD1 ROs develop typically with expression of all key retinal neurons and demonstrate expression of *ABCA4* on transcript and protein level. **A** Representative ROs derived using the BMP4-activated method of RO differentiation are displayed in the purple shaded box. PT2 and WT2 are displayed at various timepoints of differentiation spanning Day 60 to Day 220. Similarly, representative ROs derived from the IGF1-dependent method of differentiation are displayed in the green shaded box. Across all lines and protocols, clearly defined RO structures are developed by Day 120 of differentiation. By Day 180, the characteristic inner and outer segment brush border is apparent on the organoid’s apical edge and continues to develop longer structures by the final timepoint of Day 220 as evidenced by the red arrows in the final column. **B** All iPSC-derived ROs display expression of markers for REC (photoreceptor cells), SNCG (RGCs, yellow arrows), PROX1 (HCs), AP2α (ACs), PKCα (bipolar cells, white arrows) and CRALBP (Müller glia). REC^+^ cells appear to mislocalise to the central part of the ROs in a patient-specific manner. **C** ABCA4 (green) and cone-specific GT335 (red) protein expression in post-mortem retina tissue sample. The ABCA4 protein localises specifically to the POS tips of cone photoreceptor cells. ABCA4 (yellow) protein expression in nascent photoreceptor inner segments (PIS) and POS in Day 220 ROs. Staining patterns appear more diffuse throughout the PIS and POS likely due to immature disc stacking in this developmental model. The degree of observable ABCA4 fluorescence appears to correlate with the estimated residual protein levels determined via the severity of patient line genotype. AC displays the lowest intensity of fluorescence, followed by PT1, PT2 and the unaffected control WT2. **D** Representative western blotting revealed an abundance of protein at the expected size of ~250 kDa. The intensity of the bands correlated with the severity of genotype possessed by each patient case. The intensity of the ABCA4 protein bands was normalised against ACTIN (ACTB) and quantified. One-way ANOVA test revealed a significant reduction in ABCA4 expression in AC and PT1 samples. Whilst PT2 ABCA4 protein levels remained relatively close to WT2 samples. *N* = 16 ROs per sample. **** = *p*-value < 0.0001.

ROs from all iPSCs were maintained for 220 days, with development monitored by brightfield imaging (Fig. 1A) and immunohistochemistry for key markers at specific timepoints: Day 120, Day 180 (Fig. S2-S3), and Day 220 (Fig. 1B). As early as Day 60, a characteristic phase-bright neuroepithelium was observed across ROs from all iPSC lines using both protocols. This feature, common in central nervous system organoids, corresponds to the primitive neuroepithelium from which retinal precursor cells (RPCs) are derived. These RPCs continue to differentiate, giving rise to key retinal neurons: photoreceptors, retinal ganglion cells (RGCs), bipolar cells (BCs), amacrine cells (ACs), and horizontal cells (HCs), and Müller glia **(**Fig. S2, S3). From Day 180 onwards, all ROs displayed evidence of POS formation, protruding from the apical edge of the ROs (Fig. 1A).

By the final timepoint of differentiation (Day 220), all ROs displayed key retinal neurons, including photoreceptors (REC^+^), RGCs (SNCG^+^), ACs (AP2ɑ^+^), HCs (PROX1^+^), BCs (PKCɑ^+^), and Müller glia (CRALBP^+^) (Fig. 1B). The interneurons (BCs, ACs, HCs) and RGCs exhibited correct apical/basal polarity across the stratified neuroepithelium. In WT organoids from both protocols, REC^+^ photoreceptors were correctly aligned to the apical edge of the ROs. However, in STGD1 ROs, photoreceptors were found basally in addition to their expected apical localisation (Fig. 1B).

ABCA4 is most abundantly expressed in the tips of POS and is also found in the phagolysosomal membranes of RPE cells, where it functions as a flippase, facilitating the efficient removal of spent retinoids from the visual cycle [4, 5, 12]. We confirmed ABCA4 localisation to POS in post-mortem human retina samples using a cone POS-specific marker, GT335 (Fig. 1C). In ROs, ABCA4 was specifically present in photoreceptor cells but diffusely distributed across the inner and outer segments. This is likely due to nascent disc formation in photoreceptors of ROs, which are often imperfectly stacked, causing mislocalised expression [37]. In our study, differences in ABCA4 expression levels and intensity were observed across the spectrum of STGD1 ROs via immunocytochemistry, which appeared to correlate with genotype and phenotype. Patient AC possesses a complete loss-of-function (LOF) allele in trans with a missense allele, substantially reducing the amount of functional ABCA4 protein. This results in an enhanced phenotype reported as borderline cone-rod dystrophy (CRD) [38]. Patients PT1 and PT2 are both monoallelic late-onset cases of STGD1 with the same complex allele, the most severe one being c.5461-10 T > C, affecting the exon 39 splice acceptor site. This variant causes skipping of exon 39 and/or exon 40. The hypomorphic variant c.5603 A > T is located in exon 40. PT1 photoreceptors appear to express slightly less ABCA4 protein compared to PT2 (Fig. 1D**, full blot supplementary)**. This suggests that the missing allele in PT1 may be more severe than in PT2. Notably, PT2 displayed ABCA4 levels similar to WT2 photoreceptors. Importantly, these results demonstrate the ability of ROs to recapitulate ABCA4 expression and protein abundance patterns consistent with the STGD1 genotype-phenotype correlation matrix [39].

### Photoreceptor mislocalisation occurs in a disease and photoreceptor cell-specific manner

**Fig. 2:**
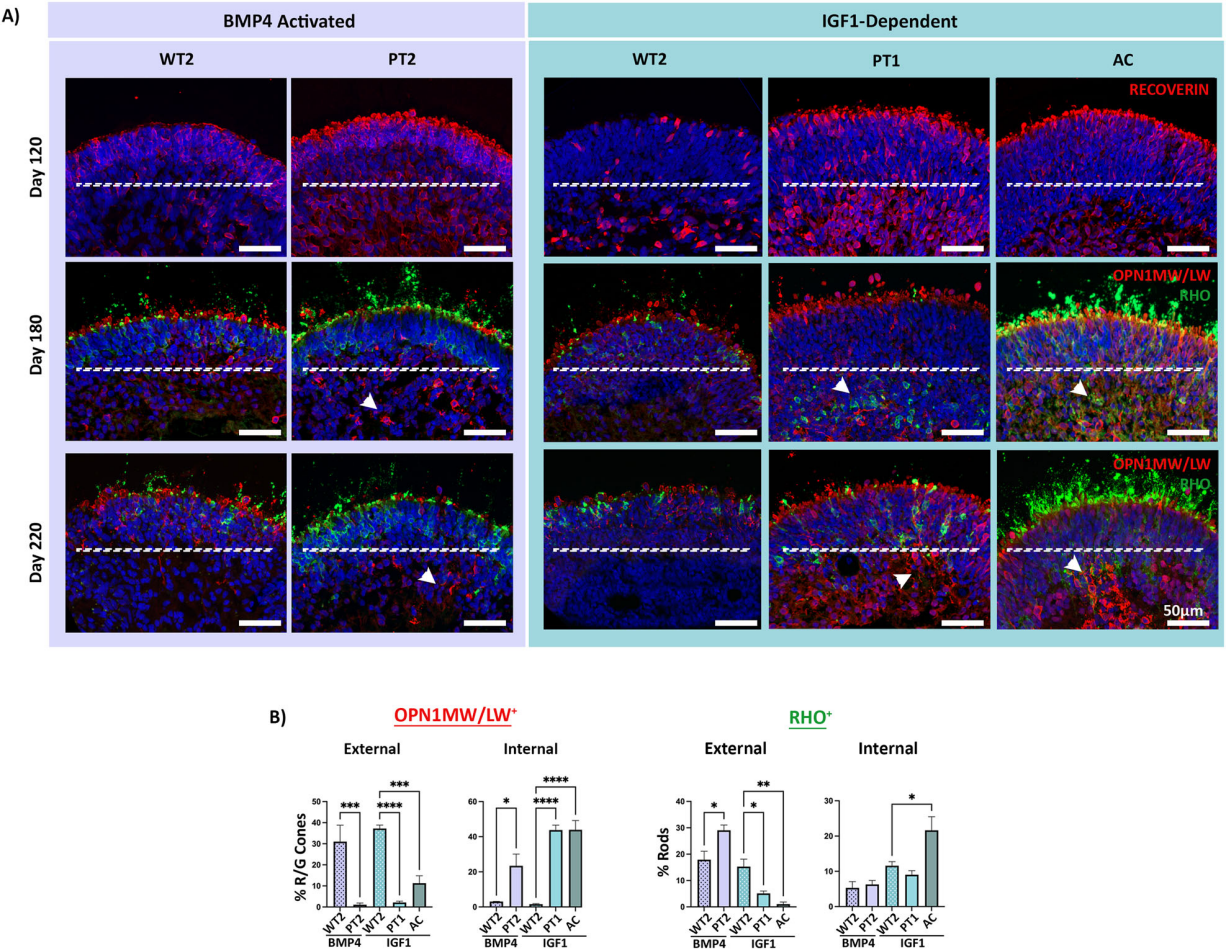
STGD1 ROs display a patient-specific photoreceptor mislocalisation that positively correlates with disease severity. **A** At Day 120, basally located REC^+^ photoreceptors are apparent in both patient and control ROs, as expected during this stage of retinogenesis. This phenotype persists at Day 180 in a patient-specific manner with the majority of photoreceptors having migrated to the apex (above white dashed line) as expected in WT2. OPN1MW/LW^+^ red/green cones being the most affected (white arrowheads). AC and PT1 ROs also display RHO^+^ rod mislocalisation. There is evidence of correctly aligned photoreceptors on the RO’s apical edge alongside positively stained OS in patient ROs also (shown above the dashed line) suggesting not all photoreceptors are affected. **B** Mislocalised OPN1MW/LW^+^ and RHO^+^ photoreceptors were quantified at Day 220 by counting the number of externally and internally positioned photoreceptor nuclei. External/Internal boundaries are defined by the white line across the central region of the RO. A significant decrease in external OPN1MW/LW^+^ cones in PT2, PT1 and AC was observed when compared with their respective WT2 controls. In contrast, internal OPN1MW/LW^+^ cones displayed a significant increase in PT2, PT1 and AC ROs. Significance was highest for the AC ROs which displayed the most enhanced phenotype. External RHO^+^ cells displayed a higher number of RHO^+^ cells in PT2 when compared with WT2, whilst AC ROs showed a significant decrease in externally aligned RHO^+^ cells. Statistics: One-way ANOVA. *N* = 5 ROs per triplicate - comparing only within protocol groups (e.g., BMP4-activated PT2 vs WT2). * = *p*-val 0.05, ** = *p*-val 0.01, *** = *p*-val 0.001, **** = *p*-val 0.0001.

As early as Day 120, a patient-specific phenotype emerged in the ROs, specifically by REC^+^ photoreceptor cells. These positively stained cells were more abundant in the cores of STGD1 ROs compared to wild type (Fig. 2A). Normally, few cells are expected in this region at Day 120, given the early stage of retinal development. However, the intensity of positively stained cells in the central regions of the organoid appeared to correlate with disease severity in patient samples. Among these, AC and PT1 ROs exhibited the most pronounced defect, followed by PT2. This phenotype persisted at two later timepoints (Day 180 and Day 220), when photoreceptors are more mature and can be distinguished as rod or cone photoreceptors using RHO and OPN1MW/LW markers, respectively.

To assess the genotype-phenotype correlation in the severity of this defect, we analysed photoreceptors located in the centre (internal photoreceptors) and on the apical edge (external photoreceptors) of the ROs at day 220 of differentiation. We quantified the OPN1MW/LW^+^ and RHO^+^ cells in an unbiased manner using a pre-designed MATLAB script (Fig. 2B). OPN1MW/LW^+^ cone photoreceptors exhibited the highest degree of mislocalisation in patient sample ROs, with a notable reduction in external cell quantity and an increase in centrally located cells. In protocol-matched wild-type controls, OPN1MW/LW^+^ cells were predominantly found along the apical edge as expected. A percentage of RHO^+^ rod photoreceptors also displayed basal mislocalisation, but this defect was observed only in ROs of patients with the most severe clinical phenotypes, namely AC and PT1. Our quantification algorithm specifically selects photoreceptor nuclei, ignoring more variable structures, like POS, across samples. With this method, a notable decrease in the number of external rod nuclei was observed, mirrored by an increase in the number of internally located nuclei in both AC sample. In contrast, rods in PT2, which had milder clinical phenotypes, were localised along the apical edge of the organoid, similar to wild-type ROs. This aligns with PT2’s later-onset symptoms and preservation of central vision via foveal sparing. These findings suggest that photoreceptor mislocalisation occurs in a ‘dose and photoreceptor cell-specific’ manner related to the expected residual ABCA4 protein, reflecting the genotype-phenotype correlation described for *ABCA4*-associated retinopathy.

### Upregulation of stress response in STGD1 photoreceptors

**Fig. 3:**
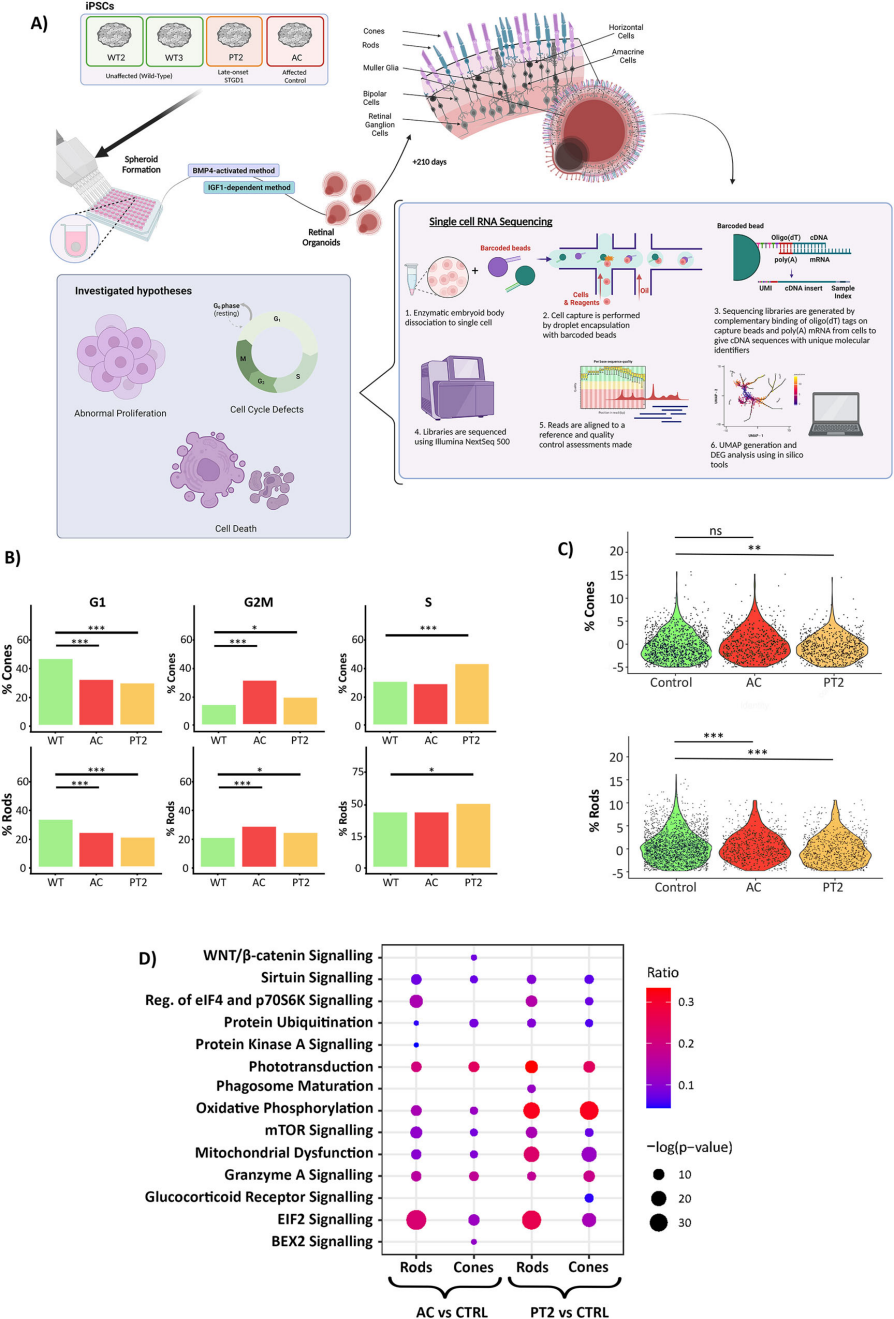
scRNA-Seq shows pathomechanism of photoreceptor mislocalisation to be likely secondary to activation of stress-response in STGD1 ROs. **A** Schematic depicting the scRNA-Seq experimental process. Briefly, ROs are derived from iPSCs and matured to Day 200 where all retinal neurons are expected to be developed. scRNA-Seq library prep is conducted on RNA transcripts from dissociated single cells of the STGD1 ROs and barcoded mRNA transcripts are sequenced using Illumina NextSeq 500 platform. **B** An analysis of cell cycle progression in STGD1 ROs. Histograms display the 3 phases of the cell cycle (G1, G2M and S phase) in both cone and rod photoreceptors of PT2, AC and WT ROs (WT = WT2 and WT3 ROs pooled). Cone and rod photoreceptors in both PT2 and AC ROs show reduced numbers of cells in G1 phase of cell cycle, and instead show increased cell numbers in the intermediatory phases S and/or G2M phase. Fisher’s Exact test was performed to measure the variance in abundance of cells in different phases of the cycle. * = p-val 0.05, *** = p-val 0.001. **C** An analysis of apoptotic gene expression in STGD1 ROs reveals elevated expression of apoptosis-related genes in PT2 cones when compared with WT control. In rods, there appears to significantly lower expression of apoptosis-related genes in both PT2 and AC ROs. **D** DEG analysis revealed several affected disease pathways in STGD1 ROs. The dot plot represents the most significantly altered pathways ranging from 10-30% (blue to red gradient) of overall genes differentially expressed between patient and control cone and rod photoreceptors. The significance of affection is depicted as the overall size of the dot ranging from a -log p-value range of 10-30. Common pathways affected include EIF2 signalling, oxidative phosphorylation, mitochondrial dysfunction and granzyme A signalling which are all involved in stress response. Phototransduction was also significantly altered across all photoreceptors. Figures were created using BioRender.com.

To underpin the mechanisms behind photoreceptor mislocalisation in STGD1 ROs, we generated scRNA-seq datasets from both mild late-onset (PT2) and severe (AC) STGD1, controlling for variability in differentiation protocols with wild-type (WT2 and WT3) derived ROs (Fig. 3A).

We first developed an integrated UMAP to visualise the cell populations present in our RO model (Fig. S4A). This clustering technique groups cells with similar gene expression patterns together, which can then be annotated using known gene expression signatures from the literature for specific cell types in that tissue. Using this strategy, we identified 17 transcriptomically distinct cell clusters in the ROs. These clusters included all key neuronal cell types expected in the retina, such as cone and rod photoreceptors, bipolar cells, RGCs, ACs, HCs, Müller glia, and RPE. Additionally, extra-retinal tissues were observed, with clusters corresponding to ocular surface epithelium (OSE), fibroblasts, astrocytes, and lens cells. Progenitor cell clusters were also present in our RO samples, corresponding to late RPCs and the transient neurogenic progenitors known to give rise to RGCs (T1), HCs and ACs (T2), and bipolar or photoreceptor cells (T3), as previously identified and reported [40]. These results align with the characterisation of ROs of recent in vitro toxicology scRNA-Seq studies from our group [41, 42], as well as scRNA-Seq studies on ROs from others [40, 43]. Furthermore, ABCA4 expression was found to be restricted to and overlapping with the cone and rod photoreceptor clusters, as anticipated (Fig. S4B).

Several hypotheses were tested to address the photoreceptor mislocalisation defect:

1. Cell cycling defects.
2. Abnormal photoreceptor proliferation resulting in the accumulation of immature cells in the centre of ROs.
3. Photoreceptor cell death preventing their maturation and migration to the apical side of ROs.

Whilst we did not find significant differences when investigating the differential proliferation of photoreceptor cells within each cohort (data not shown), we did note some differences in cell cycle stages across photoreceptors in STGD1 photoreceptors compared to each other and controls (Fig. 3B). Cone and rod photoreceptors in the AC organoids expressed significantly more markers associated with the G2/M phase of the cell cycle compared to controls, while expressing significantly fewer markers associated with the G1 phase, where photoreceptors at Day 200 are expected to arrest. Similarly, PT2 cone and rod photoreceptors expressed fewer markers associated with the G1 phase but significantly more in the G2/M and S phases of the cell cycle.

Evidence has shown that cellular migration in developing stratified neuroepithelium is highly correlated with phases of the cell cycle, a process known as interkinetic nuclear migration (IKNM) [44]. It is understood that cells in the S-phase occupy the basal positions of the neuroepithelium. In the G2/M phase, the cells begin their ascent to the apical positions of the neuroepithelium [45]. A relatively high percentage of rod and cone photoreceptors in AC ROs are in the G2/M phase, suggesting they are still in the process of IKNM, although a significant proportion are retained at the basal position, indicating that impaired IKNM is likely not the causative factor. Similarly, a large proportion of rods in PT2 ROs are in the S phase of the cell cycle but localised on the apical side of ROs, again suggesting that impaired IKNM is likely not the causative factor. Using the expression of genes involved in the induction and progression of apoptosis (Fig. 3C) we were able to show similar or lower percentages of apoptotic photoreceptor cells in patient ROs compared to WT control. To investigate this further, we carried out CASP3 immunofluorescence studies, and despite a slight increase in CASP3^+^ cells in immunocytochemistry correlating with disease severity in STGD1 ROs (Fig. S4C), the percentage of CASP3^+^ photoreceptors was very low, indicating that CASP3-mediated cell death is not the cause of photoreceptor mislocalisation in STGD1 ROs.

The molecular cause of this phenotype appears to be multifaceted, likely resulting from degenerating tissue caused secondarily by the stress of harbouring mutated ABCA4, rather than a direct effect of dysfunctional ABCA4. This hypothesis is supported by the extensive gene dysregulation observed when investigating the differentially expressed gene (DEG) networks in photoreceptors of STGD1 ROs versus control ROs (Fig. 3D). Affected pathways with abundant DEGs included mTOR signalling, mitochondrial dysfunction, oxidative phosphorylation, granzyme A signalling, and EIF2 signalling, which are hallmarks of the cellular stress response [46, 47]. Hyperactivation of the mTOR signalling pathway has also been reported to disrupt retinal lamination in ROs [48]. Notably, phototransduction was a key affected pathway, demonstrating that ROs are a suitable model to dissect the molecular pathology of STGD1.

### Biallelic resolution of STGD1 monoallelic late-onset cases uncovered via whole genome sequencing

To resolve the monoallelicism of PT1 & PT2 cases, we opted for a WGS strategy combined with a long-read RNA sequencing at the *ABCA4* locus. As most of the coding variation in *ABCA4* has thus far been identified [49], we hypothesised that the missing allele could be either a hypomorphic variant or perhaps a non-coding variant that alters transcript expression.

**Fig. 4:**
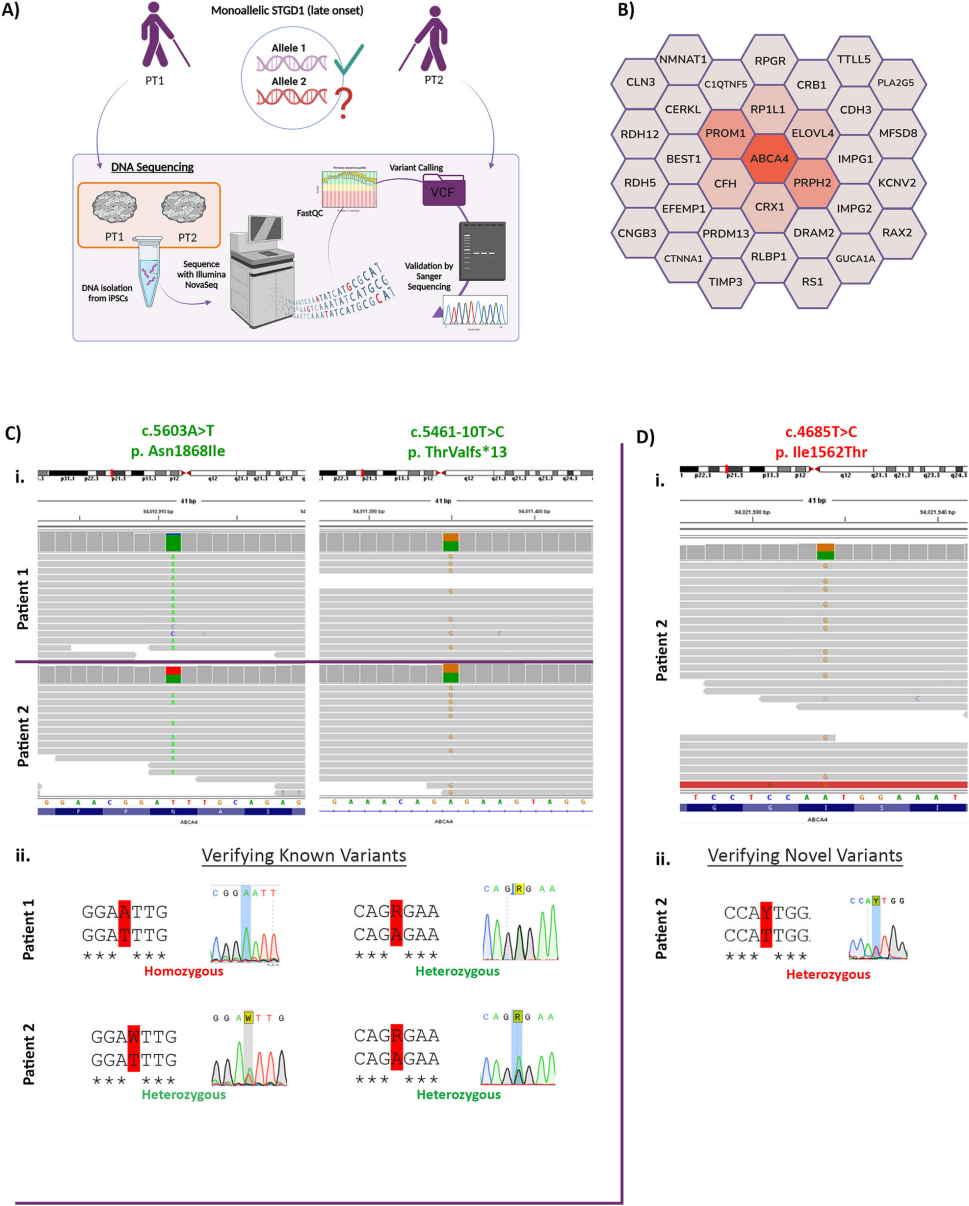
Detection of missing alleles in PT1 and PT2 cases with WGS. **A** Schematic of DNA sequencing strategy. Briefly, DNA was isolated from PT1 and PT2 iPSCs and sequenced with the Illumina NovaSeq to yield WGS datasets for the identification of missing inheritance in PT1 and PT2 samples. **B** Macular disease gene panel of 33 genes used in the filtering of WGS to identify missing variants in the monoallelic STGD1 cases included in this study. Likely to least-likely genes causative of disease phenotype are arranged spatially from the centre to periphery and colour coded red to grey respectively. **C** (i). IGV view of the c.5461-10 T > C and c.5603 A > T variant in *ABCA4*. Read depth appears to be approx. 50% in both PT1 and PT2 indicating heterozygosity of c.5461-10 T > C validating previous reports with these patients. Read depth appears to be approx. 50% in PT2 for c.5603 A > T indicating heterozygosity of this variant validating previous reports. However, almost 100% read depth in PT1 suggests homozygosity of the variant thereby providing biallelic resolution for PT1. *ABCA4* is viewed in 3’←5’ orientation on IGV and so variants are read in reverse complement. (ii). Sanger sequence traces verifying the variant identified in NGS analysis. **D** (i). IGV view of the c.4685 T > C variant in *ABCA4*. Read depth appears to be approx. 50% in PT2 indicating heterozygosity of this variant, potentially resolving the missing allele in PT2 case. (ii). Sanger sequence traces verifying the variant identified in NGS analysis. Figures were created using BioRender.com.

WGS of PT1 and PT2 DNA (Fig. 4A) resulted in high-quality sequence reads with sequence read length averaging approximately 150 bp. Variant calling was facilitated through alignment with the hg38 reference genome. The sequence reads passed the FastQC report with high PHRED scores of 35.1 and 35.55 for PT1 and PT2 respectively. This enabled us to successfully call potential candidate variants in PT1 and PT2 as well as confirm the known *ABCA4* variants.

The known variants observed in the WGS of both PT1 and PT2 included the c.5461-10 T > C intronic variant and the c.5603 A > T hypomorphic variant in *ABCA4*, both reported in heterozygous state. These variants were initially identified by our collaborators at RUMC using Haloplex sequencing, a high-throughput, targeted sequencing approach for the *ABCA4* gene, which provides high read coverage and enables the multiplexing of many patient samples to uncover variants causing monogenic diseases [50, 51].

To uncover the missing allele, we filtered our WGS datasets to include genes known to cause inherited maculopathies (Fig. 4B), however we did not detect any promising causative variants in genes other than *ABCA4*. We also extended the panel to include genes involved in inherited retinopathies, but this did not reveal any substantial findings. We did however confirm the presence of known intronic variant c.5461-10 T > C in both PT1 and PT2 in heterozygous state as previously reported (Fig. 4Ci). This was further validated in the Sanger sequencing traces which corresponded to overlapping peaks of the nucleotides T and C demonstrating heterozygosity at that allele (Fig. 4Cii). We also confirmed the presence of hypomorphic allele c.5603 A > T in the same manner, which showed heterozygosity in PT2 as demonstrated by overlapping peaks of A and T nucleotides. Surprisingly, we observed the c.5603 A > T variant in homozygous state both in WGS and Sanger sequencing in PT1. This defined PT1 full genotype as **Allele 1**: c.[5461-10 T > C;5603 A > T] and **Allele 2:** c.5603 A > T.

In PT2, we identified an *ABCA4* variant that was not detected in the first round of DNA sequencing by our collaborators. The missense variant c.4685 T > C was present in heterozygous state in both the WGS BAM files and validated with Sanger sequencing (Fig. 4D). This variant has been reported previously on ClinVar with contradicting reports of pathogenicity, resulting in the reporting of this variant as a variant of uncertain significance. This is likely due to the observation that c.4685 T > C in homozygous state is not causative of disease and MAF frequencies are observed in the general population with ExAC frequencies in European cohorts at 0.001305 versus allele frequencies in STGD1 cohorts as 0.00153. The c.4685 T > C variant subsequently was classified as a rare hypomorph, such that its penetrance is only apparent in combination with a null allele in trans [49]. On this basis, we have determined that PT2 is biallelically resolved with the genotype as **Allele 1**: c.[5461-10 T > C;5603 A > T] and **Allele 2:** c.4685 T > C.

Interestingly, a recent publication revealed the potential for *trans*-acting modifiers of STGD1, namely in the *PRPH2* gene [52]. When investigated, we found that both our PT1 and PT2 cases harboured the EKG haplotype in *PRPH2* (Fig. S5). This haplotype influences the penetrance of the common hypomorph c.5603 A > T, p.(Asn1868Ile) and could explain why PT1’s phenotype is more severe as a homozygote than PT2 despite them both displaying late-onset STGD1.

### LRS confirms the resolved genotypes of PT1 & PT2

**Fig. 5:**
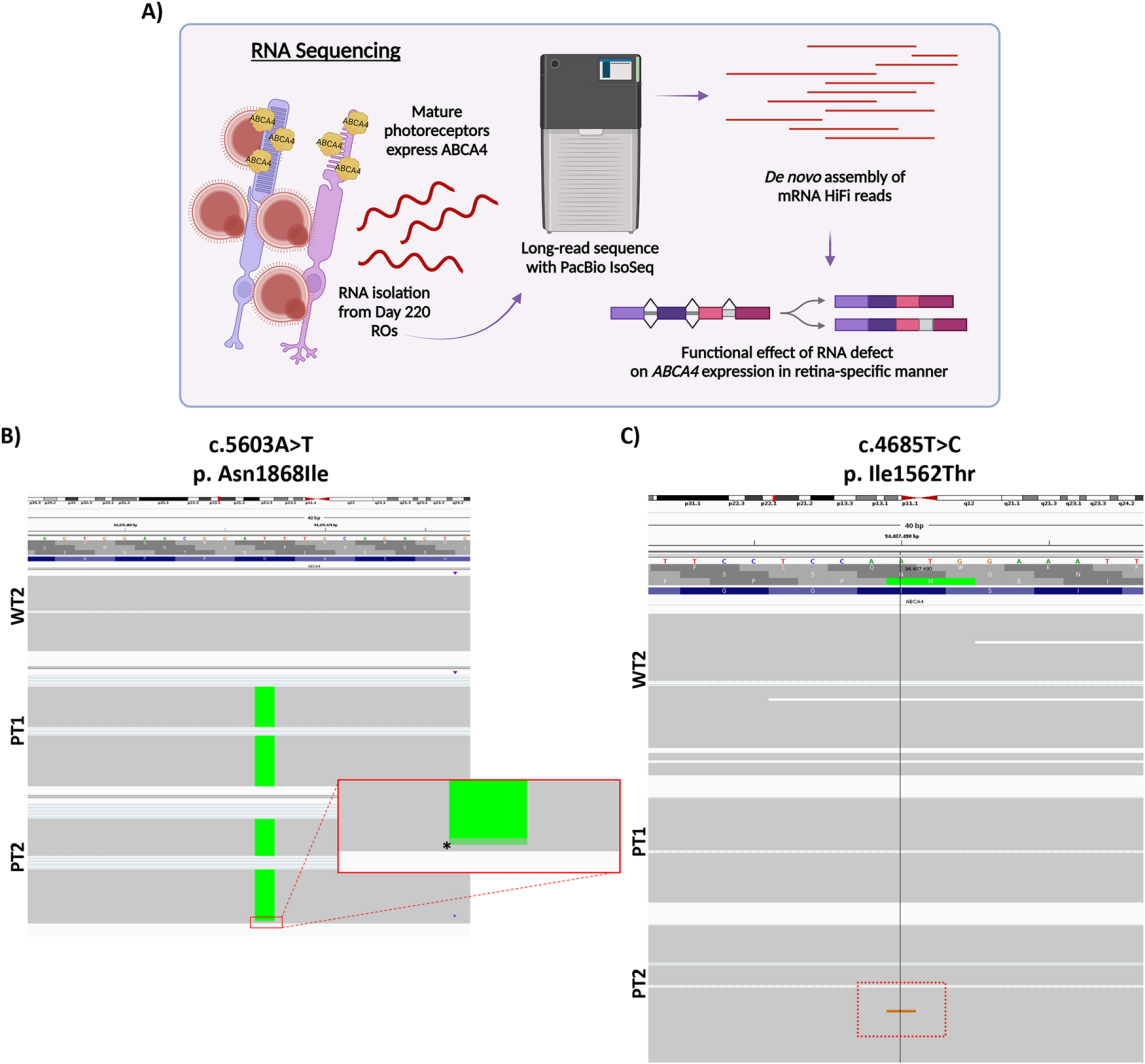
Validation of WGS-identified variants in LRS. **A** Schematic of LRS strategy for assessing RNA defects in PT1 and PT2 cases. RNA transcripts are isolated from 220-day old ROs to ensure capture of tissue-specific *ABCA4* isoforms. **B** HiFi reads displayed in IGV browser for LRS data derived from Day 220 ROs. WT2 displays no defect, whereas PT1 and PT2 display the A > T variant change. The green boxes correspond to the nucleotide A, which on the reverse strand is T. The variant is homozygous in PT1 and appears to be dominantly expressed in PT2 despite its heterozygous status, except for one read (red box) which is unaltered. **C** HiFi reads displayed in IGV browser for LRS data derived from Day 220 ROs. The c.4685 T > C variant is present in one read of PT2 transcripts. The orange read within the red box shows a nucleotide change to ‘G’, which in the reverse strand is ‘C’ consistent with the reported variant. The reduced incidence of reads containing this variant are likely due to transcript degradation or perhaps ASE. Figures were created using BioRender.com.

RNA was isolated from Day 220 ROs to obtain tissue-specific *ABCA4* transcripts and sequenced using PacBio Iso-Seq. Transcript reconstruction from HiFi reads was conducted to define different transcript models of *ABCA4*, as shown in Fig. 5A. The c.5603 A > T, p. Asn1868Ile mutation was observed in IGV for both PT1 and PT2 (Fig. 5B). Sequencing studies described above have shown that PT1 is homozygous for c.5603 A > T, while PT2 is heterozygous. Despite this, all *ABCA4* HiFi reads contained this mutation, with the exception of just one read, as indicated by the red box in Fig. 5B. This intriguing observation suggests the possibility of allele-specific expression (ASE) in PT2.

We made another intriguing observation while attempting to validate the newly discovered variant c.4685 T > C, p.(Ile1562Thr) in the HiFi reads from the LRS. As shown in the IGV browser (Fig. 5C), the c.4865 T > C variant in just one read of the *ABCA4* transcripts of PT2, but not in either WT2 or PT1 transcripts. The rest of the reads contained the reference nucleotide. It was noted during quality checks that RNA sequences from PT2’s ROs exhibited signs of degradation, yet sequenced isoforms consistently displayed the c.5603 A > T variant. We hypothesised that perhaps the reduced incidence of reads containing the c.4685 T > C variant could stem from transcript-specific degradation possibly due to misfolding or influences from *cis*- or *trans*-acting modifiers affecting *ABCA4* allele expression, in addition to incomplete nonsense-mediated decay suppression during RO culture. Coupled with ASE, this could explain why most reads contained the c.5603 A > T variant despite PT2 being heterozygous for this variant in WGS and Sanger data.

### STGD1 ROs are capable of recapitulating functional splicing defects

As WGS was sufficient in uncovering the missing alleles in PT1 & PT2 cases, and no other RNA defect was observed in the LRS data to account for STGD1 phenotypes, we utilised the data to gain functional insights into the c.5461-10 T > C variant in *ABCA4*. As opposed to short-read sequencing, LRS gives unique insights into gene expression patterns with full-length isoform resolution. Our aim was to investigate if the STGD1 ROs could recapitulate the splicing pattern of this variant reported in the literature [53].

**Fig. 6:**
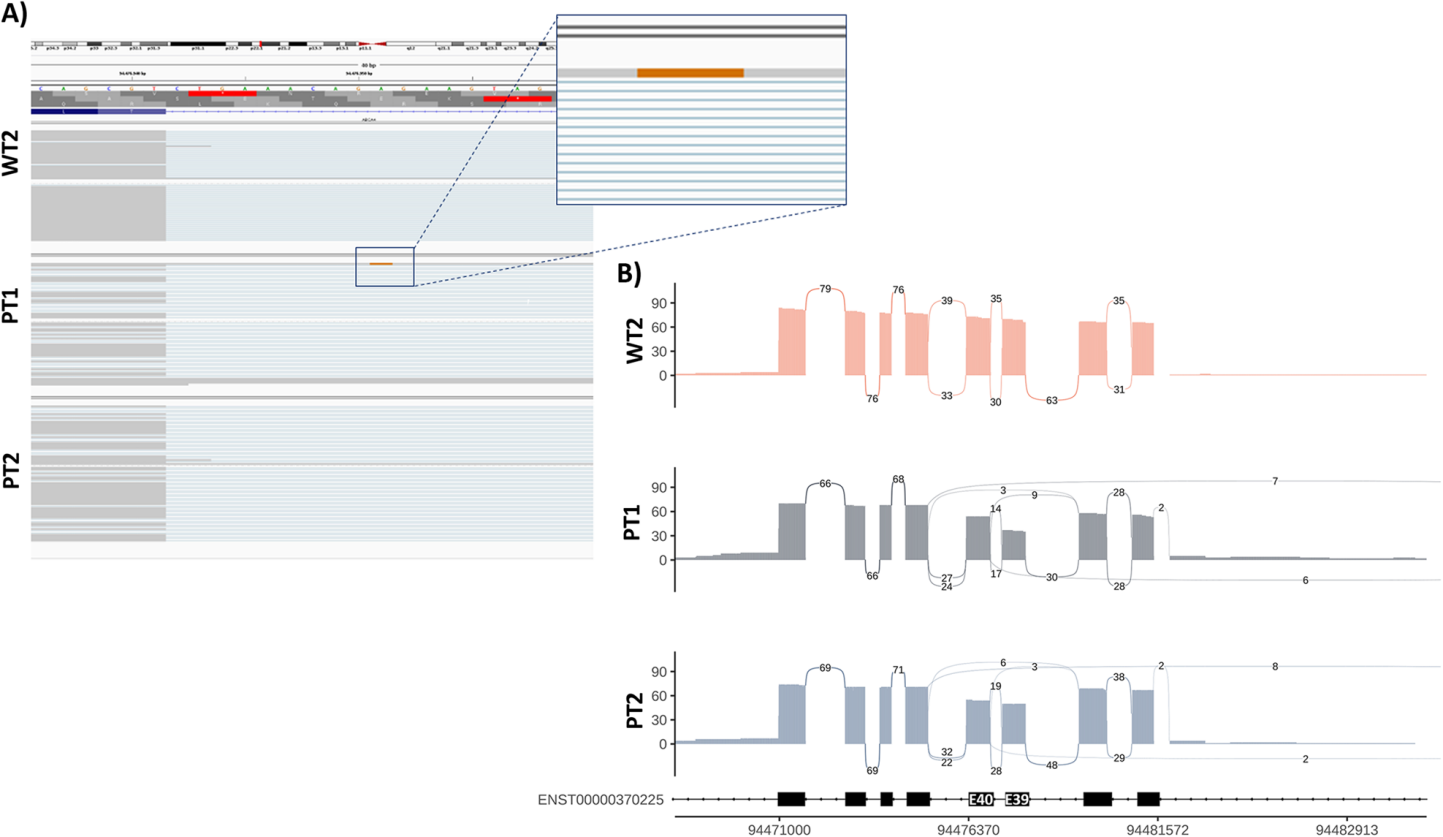
Functional effects of c.5461-10 T > C intronic variant on *ABCA4* expression in STGD1 ROs. **A** HiFi reads displayed in IGV browser for LRS data derived from Day 220 ROs. Intronic variants are not typically observed in sequenced reads unless the intron is retained. PT1 has evidence of one intron-retaining read containing the mutation in question (brown nucleotide in navy box). The brown nucleotide corresponds to G, which is C on the reverse strand. **B** Sashimi plot indicating the number of long reads (PacBio HiFi reads) supporting the skipping of exon 39 or exons 39 + 40 (associated with the c.5461-10 T > C variant) in *ABCA4* from WT2, PT1 and PT2.

We were not expecting to see the 5461-10 T > C, p.[Thr1821Valfs*13,Thr1821Aspfs*6] variant in the HiFi sequence reads from any patient or control as this variant falls within an intronic region. However, in the IGV browser, we were able to observe one read in PT1 that had retained this intron and harboured the mutation as described **(**Fig. 6A**)**. The brown read corresponds to the nucleotide G in that position, which is C on the reverse strand (i.e., T > C change), further validating the presence of this mutation in RNA transcripts of PT1.

c.5461-10 T > C has been shown to induce the skipping of exon 39 and/or exon 40 of *ABCA4* [53]. To visualise this, the splicing events from PT1 and PT2 were represented in a Sashimi plot (Fig. 6B). This plot indicates the number of reads supporting exon inclusion/exclusion, which is then used to quantify the degree of exon skipping occurring. Transcripts from the WT2 ROs displayed a PSI value of 1 as expected, but the patient organoids displayed lower values. For exon 39, PT1 exhibited a PSI value of 0.55, and PT2 0.71. For exon 40, PT1 exhibited a PSI value of 0.83 and PT2 a value of 0.79. The higher degree of PSI in PT2 confirms that the splicing effects of the c.5461-10 T > C variant are not as penetrant in PT2 as previously hypothesised. The effects of c.5641-10 T > C at RNA and protein levels (based on PT1 full-length transcript models) can be observed in Fig. S6. Overall, the LRS data was able to recapitulate splicing patterns of native retina and reveals the variable effect the c.5461-10 T > C variant on *ABCA4* expression, in accordance with the existing literature.

## Discussion

Our study is the first to track STGD1 across multiple genotypes in an in vitro model throughout retinogenesis, spanning from Day 60 through to Day 220 of differentiation. We demonstrate that the STGD1 RO model expresses *ABCA4* on transcript and protein level, and moreover is capable of recapitulating retina-specific *ABCA4* splicing patterns in vitro.

Using an iPSC-derived STGD1 model, we identified a quantifiable defect of photoreceptor mislocalisation that intensifies in direct correlation with the genotypic severity at the *ABCA4* locus. We observed a heightened vulnerability of cone photoreceptors to ABCA4-associated disease, consistent with previous findings in mouse models of STGD1 [54] and in retinas of STGD1 patients [55]. Interestingly, this defect manifested even in the absence of functional RPE tissue, challenging the traditional understanding of STGD1 pathomechanism. Conventionally, the disease involves A2E-laden POS shed daily and phagocytosed by RPE cells, leading to the formation of lipofuscin aggregates and subsequent RPE cell loss, compromising overlying photoreceptors. However, the presence of ABCA4 expression in RPE cells (Lenis et al. [5]) and the discovery of autonomous lipid handling defects in iPSC-derived RPE from STGD1 patients [56] suggest a more complex scenario. In vivo, it is likely that ABCA4 deficiency in both neural retina and adjacent RPE cells creates a cytotoxic environment within the fovea, where there is a high turnover of retinoids daily.

Our data demonstrate that cones are more susceptible to ABCA4-associated degeneration than rod photoreceptor cells. Previously, in an in vivo *Rho*^*-/-*^ *Abca4*^*-/-*^ mouse model, it was found that cone cells generate substantially more A2E per mole of retinoid compared to their Abca4-deficient rod counterparts [54]. Additionally, fewer lipofuscin deposits were observed in the RPE of *Rho*^-/-^ *Abca4*^*-*/-^ mice, suggesting that rod photoreceptors are more efficient at metabolising retinoids even with ABCA4 dysfunction. This might explain why the majority of ABCA4-associated retinopathy is largely restricted to macular regions despite the uniform expression of ABCA4 across all photoreceptors, except in the context of two biallelic null alleles. More recently, cone sensitivity was demonstrated in humans under both photopic (favouring cone photoreceptors) and scotopic (favouring rod photoreceptors) conditions in the central retina using chromatic pupil campimetry as a measure of functional degeneration of photoreceptors. This study showed no changes to rod function in the central retina of STGD1 patients, whereas cone function was significantly altered [57].

Our findings support the theory that photoreceptor cell degeneration may precede RPE cell loss, challenging the classic STGD1 pathophysiology paradigm. This phenotypic defect occurs autonomously in a model devoid of functional RPE. This observation aligns with in vivo findings using adaptive optics scanning laser ophthalmoscopy in the retinas of STGD1 patients, which demonstrated the preservation of RPE cells but a loss of cone photoreceptors within the foveal regions [55].

This study notably used two distinct RO differentiation protocols due to challenges in differentiating all iPSC samples using the BMP4-activated method. In controlled differentiations, a low dose of BMP4 on Day 6 guides iPSCs towards neural ectoderm, establishing an early retinal fate through modulation of SMAD signalling [58]. This promotes eye field transcription factors such as *PAX6, RAX* and *LHX2*, while BMP4 interactions with fibroblast growth factor and retinoic acid further supports retinal fate determination. This approach enables a more directed lineage commitment, higher differentiation efficiency and uniform cell populations across resulting ROs. However, variability in iPSC response to BMP4 for retinogenesis is reported [59] and poorly understood, though potentially due to genetic and epigenetic differences or reprogramming variabilities in iPSC lines. Conversely, the IGF1-dependant method does not use external morphogens for retinal lineage direction. Instead, single-cell dissociation and forced aggregation to embryoid bodies enable spontaneous differentiation via endogenous cues [60, 61]. IGF1 supports cell growth and survival through PI3K/Akt pathways during neuroretinal development [62, 63]. While this method better mimics early eye development *in utero*, it often results in mixed cell populations, including extraretinal cells [41].

Despite differences in RO differentiation protocols, all follow a similar developmental trajectory, ultimately recapitulating human retinogenesis. An important study compared ROs derived through an established spontaneous differentiation protocol, with the addition of various exogenous factors reported to enhance retinal development [64]. In this study, early-stage ROs were broadly similar, and as they matured, they could be grouped into three distinct stages, characterised by features such as retinal neuron localisation and the presence of an apical brush border at the final stage (stage 3). The main advantage of RO cultures is their ability to generate semi-mature photoreceptors. For ABCA4 studies, the level of maturation exhibited by both BMP4-activated and IGF1-dependant protocols, with defects quantified only at ‘Stage 3’ ROs in both protocols, is sufficient to assess photoreceptor defects and ABCA4 levels when normalised against protocol-specific controls.

Whilst we were unable to uncover the underlying mechanism of photoreceptor mislocalisation in STGD1-ROs using our scRNA-seq data, our study revealed significant dysregulation in genes associated with phototransduction and stress-response pathways. Specifically, pathways such as mTOR signalling, mitochondrial dysfunction, oxidative phosphorylation, and others showed notable upregulation. These pathways are often implicated in unfolded protein response [46, 47]. Interestingly, recent scRNA-seq analysis of iPSC-derived RPE cells from STGD1 patients also reported similar abnormalities in mitochondrial structure and function, along with dysregulation of genes involved in UPR [65]. This supports the hypothesis that mutated ABCA4 creates a stressful environment in the tissue, leading to cellular degeneration in affected areas such as photoreceptors and RPE cells. This consolidation of findings emphasises the role of *ABCA4* variants in generating tissue stress, ultimately contributing to cellular degeneration in the retina.

Our findings regarding cone mislocalisation, stress-response dysregulation, and genotype-phenotype correlations were robust across both protocols. This consistency demonstrates that the observed STGD1-specific phenotypes are intrinsic to the disease model, rather than being artefacts of the differentiation process. Although each method provides unique insights; BMP4 for directed lineage commitment and IGF1 for broader cellular heterogeneity – our primary observations of photoreceptor mislocalisation and altered ABCA4 expression were comparably represented with the addition of protocol-specific controls to facilitate meaningful normalisation.

The need for a suitable model to study ABCA4-associated diseases is highlighted by the current scarcity of effective therapeutic options. To advance potential treatments, a thorough understanding of the complete pathomechanism and functional implications of *ABCA4* variants is essential. Regarding deep-intronic variants, a promising approach involves antisense oligonucleotides (AONs), which target splice-altering variants by inducing the cleavage of double-stranded RNA molecules. This innovative technology has already shown efficacy in addressing several intronic variants within *ABCA4* [19, 38]. Most notably, recent studies have demonstrated significant success with the c.5461-10 T > C variant, achieving a 53% increase in correctly spliced *ABCA4* transcripts in retinal organoids treated with a variant-specific AON [66]. These advancements highlight the potential of AONs as a therapeutic strategy for ABCA4-associated diseases, providing a promising avenue for further research and development.

Central to this is the successful resolution of *ABCA4* genotypes for each STGD1 patient, which was successfully carried out herein for PT1 and PT2. Our data indicate that the unresolved inheritance patterns in both patients were clarified through missense variants in the coding regions of *ABCA4*. We hypothesised that the missing variations might be either hypomorphic alleles or deep-intronic variants/RNA defects, which are more challenging to detect with traditional sequencing methods. Consequently, we employed specialised RNA sequencing technology. PT1’s genotype was resolved via WGS and Sanger sequencing, which confirmed the homozygosity of the c.5603 A > T variant. On the other hand, PT2 was found to carry a rare hypomorphic variant, previously reported by Lee and colleagues. With the resolution of these cases and the absence of a protein-truncating variant on the second allele, we would expect 50% normal expression of ABCA4 across PT1 and PT2 samples. However, we observe more protein expression in PT2 than expected. We suspect that this could be a consequence of the rare hypomorph c.4685 T > C and ASE, stabilising the expression of *ABCA4* on the *trans* allele, where we have observed the PSI of c.5461-10 T > C-induced exon skipping is higher for exon 40 and thus less detrimental to the protein, in predictive models from the LRS RNA data. However, more investigation is required to fully understand the effects of this rare hypomorph.

Hypomorphic variants are intriguing and have only recently been recognised as major contributors to ABCA4-associated retinal disease. For example, the c.4685 T > C variant is often misclassified as benign due to its high MAF in the general population, which obscures any potential enrichment in the STGD1 population. The true pathogenic effect of hypomorphic variants becomes evident only when they exist in trans with a null allele. When this occurs, these variants often exhibit a “clinically dominant” effect, meaning the resulting phenotype is primarily influenced by the hypomorphic variant, irrespective of the variant in trans*.* This phenomenon results in a phenotype that is highly dependent on the hypomorph, more so than the other allele [67].

## Summary

Overall, the results generated through this research provide invaluable insights into the intricate pathology of STGD1. Our study has achieved genetic resolution for previously monoallelic cases of STGD1, enabling these individuals to participate in clinical trials for emerging treatments. Importantly, we demonstrate that genotype-phenotype correlations are possible in vitro, with variant-induced molecular pathology, making this model vital for drug development, in vitro toxicology, and further disease modelling.

The development and characterisation of the STGD1-RO model represents a significant leap forward in our understanding of this condition, allowing for a more detailed dissection of its underlying mechanisms. While this study provides robust insights into genotype-phenotype correlations in late-onset STGD1 and severe STGD1 cases, further investigation into underlying mechanisms of photoreceptor mislocalisation, altered stress-signalling pathways, inclusion of more diverse STDG1 cases and more congruent RO differentiation methods over longer time points would significantly enhance our understanding of the spectrum of ABCA4-associated pathology. The addition of iPSC-RPE cultures with the same samples in parallel would also strengthen future work. This work sets the groundwork for such studies, highlighting the potential of RO models for comprehensive disease modelling. As we continue to unravel the complexities of STGD1 through this innovative model, we advance the scientific understanding of the disease and also pave the way for the development of more targeted and effective therapeutic interventions. Ultimately, this research holds great promise for the future of STGD1 patients, offering renewed hope for improved diagnosis, treatment, and, most importantly, a better quality of life.

## Supplementary information

Suppl material

Suppl Tables

## Data Availability

scRNA-Seq data has been deposited to GEO under the following accession number: GSE236097.
