# Supplementary material for: Unravelling genotype-phenotype correlations in Stargardt disease using patient-derived retinal organoids
Source: Cell Death Dis. 2025 Feb 19;16(1):108. doi: 10.1038/s41419-025-07420-7 (PMC11840025; doi:10.1038/s41419-025-07420-7)
Supplement: Supplementary file 1 — Suppl material [file 41419_2025_7420_MOESM1_ESM.docx]

**Supplementary Information:**

**Unravelling Genotype-Phenotype Correlations in Stargardt Disease Using Patient-Derived Retinal Organoids**

**Avril Watson^1,2^**, Rachel Queen^1^, Luis Ferrández-Peral^3^, Birthe Dorgau^1^, Joseph Collin^1^, Andrew Nelson^4^, Rafiqul Hussain^1^, Jonathan Coxhead^1^, Michael McCorkindale^1^, Robert Atkinson^1^, Darin Zerti^5^, Valeria Chichagova^2^, Ana Conesa^3^, Lyle Armstrong^1,2^, Frans P.M. Cremers^6^, **Majlinda Lako^1*^**

1. ^Biosciences Institute, Newcastle University, Newcastle upon Tyne, UK^
2. ^Newcells Biotech Ltd., Newcastle upon Tyne NE4 5BX, UK^
3. ^Institute for Integrative Systems Biology, University of Valencia, Spain^
4. ^NU-OMICs, Northumbria University, Newcastle Upon Tyne, UK^
5. ^Department of Biotechnological and Applied Clinical Sciences, Università degli Studi dell'Aquila, Italy^
6. ^Department of Human Genetics, Radboud University Medical Center, Nijmegen, the Netherlands^

^* to whom correspondence should be addressed:^

^Majlinda Lako (^[^majlinda.lako@ncl.ac.uk^](mailto:majlinda.lako@ncl.ac.uk)^)^

**
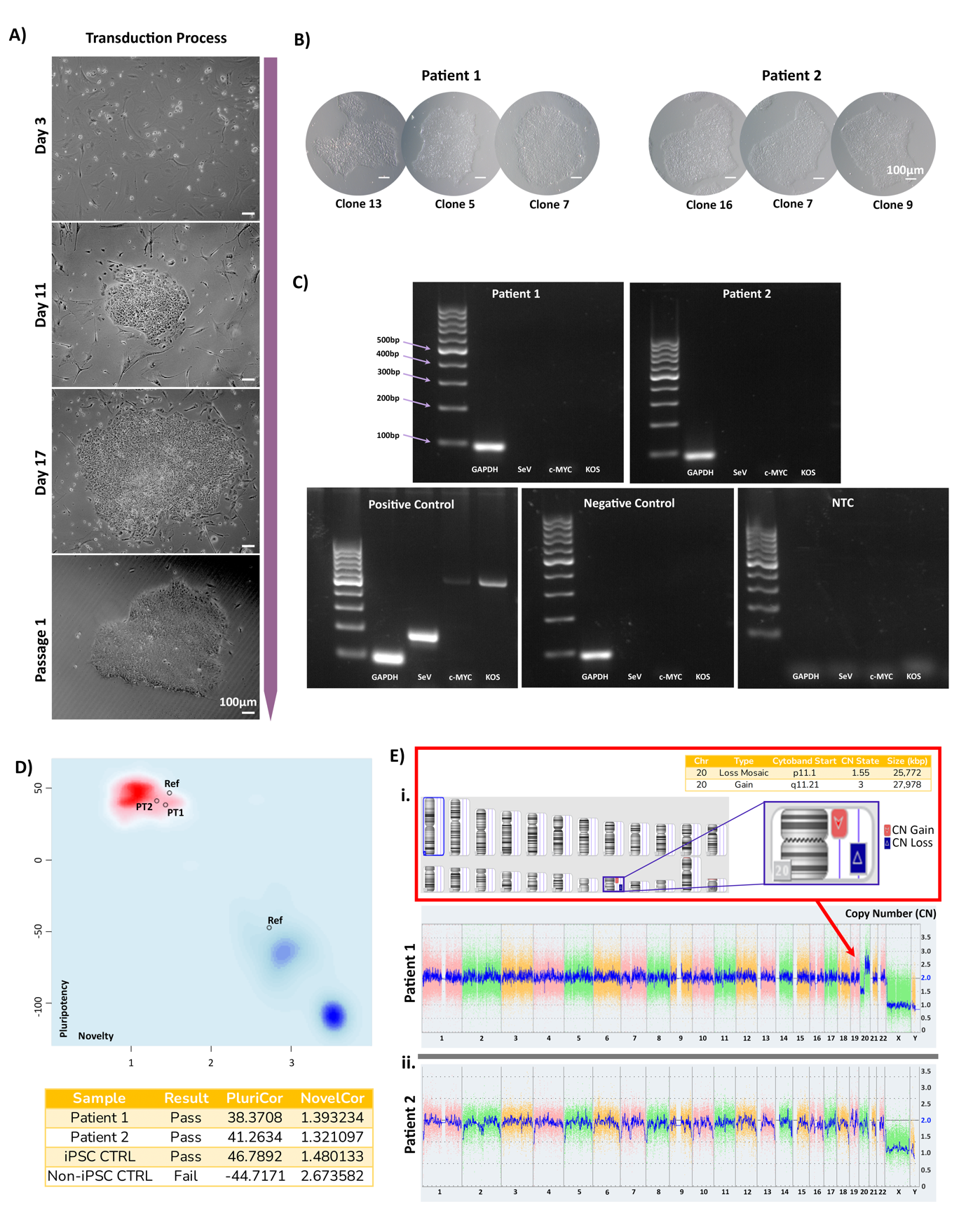
**

**Fig. S1: Cellular reprogramming and characterisation of PT1 and PT2 iPSCs.**

1. Transduction of PBMCs with CytoTune 2.0 reprogramming kit. Cells are plated with Sendai viruses on Day 1. By Day 3, there is evidence of cytotoxicity with floating cells. By Day 11, domed colonies are apparent indicating success of viral transduction. By Day 17, colonies with discrete borders, high nuclear to cytoplasmic ratio and tightly packed cells are apparent. At this point, colonies are cleared of spontaneous differentiation and feeder cells before transfer to Matrigel for adaptation to feeder free culture.
2. Three clones from both Patient 1 and Patient 2 adapted well to feeder-free conditions on Matrigel. Brightfield images of the colonies show discrete colony borders, tightly packed cells with no evidence of spontaneous differentiation.
3. Clearance of SeV vectors following the generation of iPSCs and serial passaging was achieved by passage 16. RT-PCR for SeV and SeV-transgenes revealed no corresponding bands in either Patient 1 or Patient 2 iPSCs, similarly to negative iPSC control. In the positive control, bands corresponding to *SeV* (181 bp), *c-MYC* (532 bp) and *KOS* (528 bp) were apparent. *GAPDH* (86 bp) was utilised as a loading control and was apparent in each iPSC sample tested. NTC serves as the non-template control for this experiment.
4. Pluripotency of PT1C5 & PT2C7 iPSCs was assayed using PluriTest. This assay compares the transcriptomic profile of provided samples with both pluripotent and non-pluripotent cells and tissues in a reference dataset, yielding a PluriCor score. This score is an indication of how close transcriptomic profiles are between provided samples and the pluripotent samples in the reference dataset. A novelty score is also assigned which indicates a model fit for the provided sample with the reference database. The scores are graphed on a XY scatterplot alongside controls. The red and blue background illustrate empirical distribution to the pluripotent (red) and non-pluripotent (blue) samples in the reference dataset.
5. Cells were characterised externally using the KaryoStat™ assay, which assesses the copy number variant (CNV) of SNPs in each chromosome with a reference dataset to identify any genomic instability resulting from aneuploidies, sub microscopic aberrations, and mosaic events. The signal recorded for each SNP is normalised to give a smooth signal (blue line) which gives an indication of chromosomal gains (>2) or chromosomal losses (<2). i) Genomic instability was noted in PT1 iPSCs with gain at 20q11.2 and loss at 20p11.1. ii) PT2 display no evidence of genomic instability. Both patients are male as shown by one copy of X and Y chromosomes in both karyographs.

**
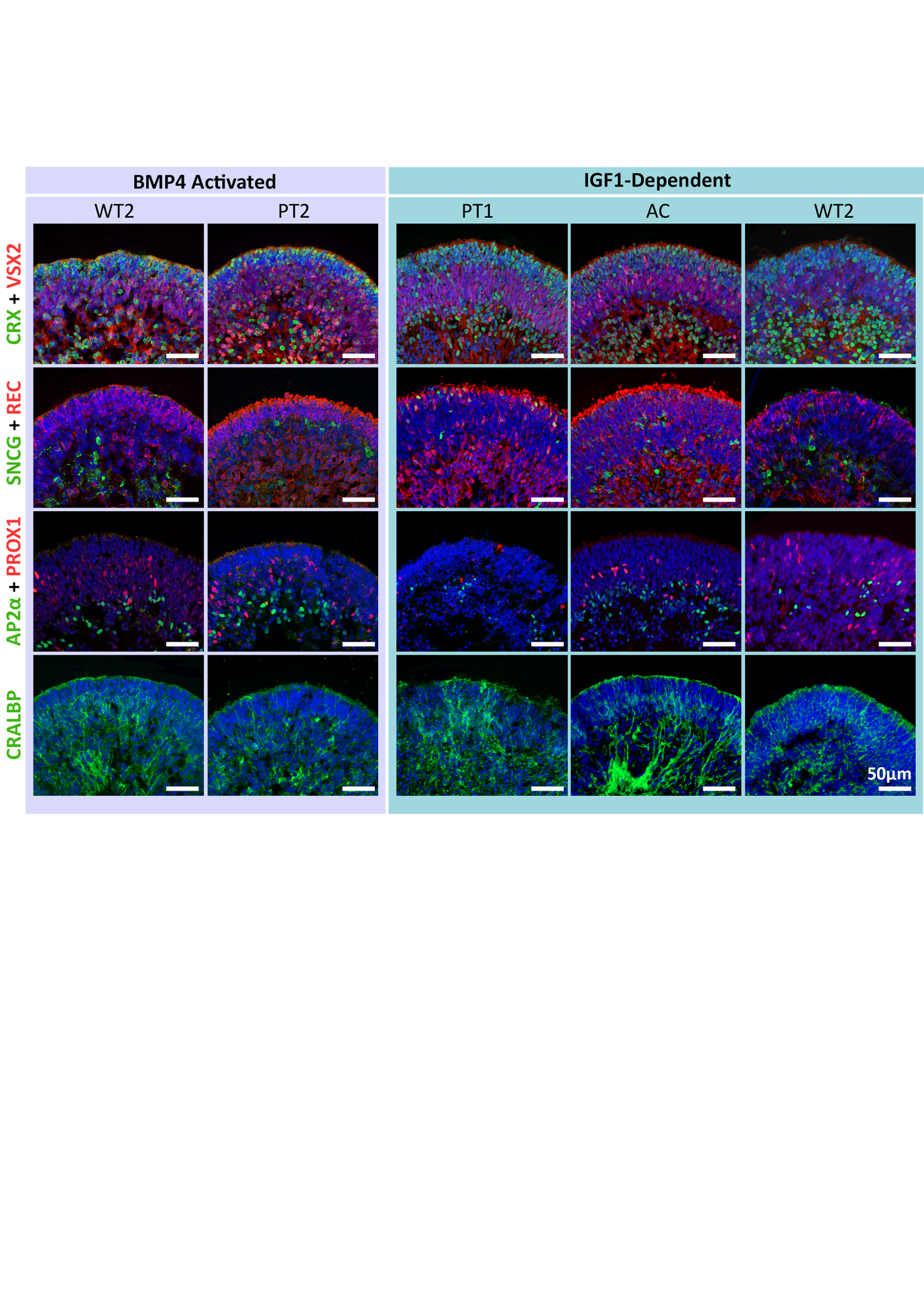
**

**Fig. S2: Characterisation of day 120 ROs by immunofluorescence microscopy.**

Despite different protocols being used, all ROs stained positively for markers of early and late neurogenesis at this intermediary stage of retinal development. Positive markers included CRX (photoreceptor precursors), VSX2 (retinal progenitor cells), REC (photoreceptors), SNCG (RGCs), PROX1 (HCs), AP2α (ACs) and CRALBP (Müller glia). This suggests each patient and control iPSC-ROs are developing as expected and are suitable for use at later developmental timepoints.

**
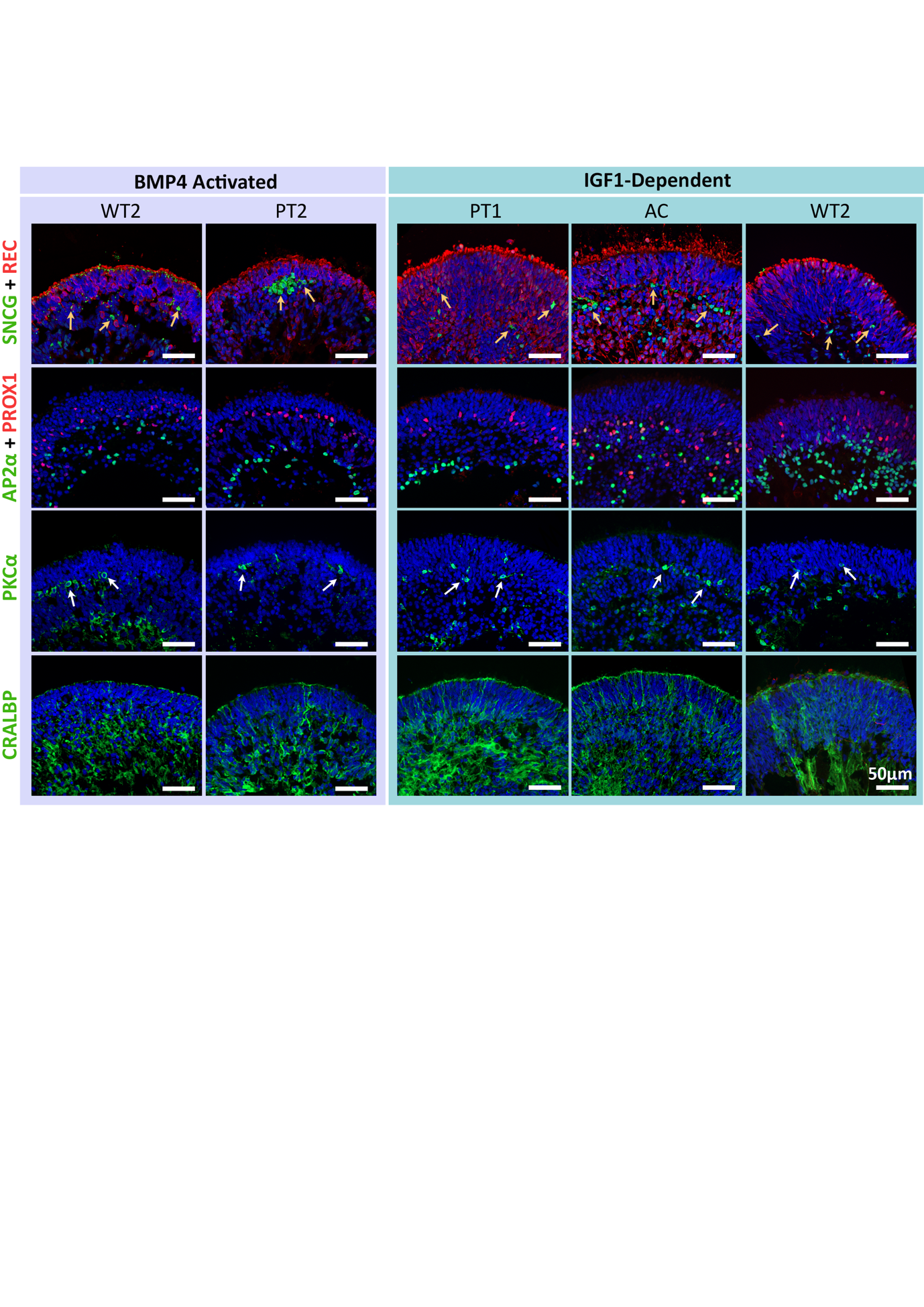
Fig. S3: Characterisation of day 180 ROs by immunofluorescence microscopy.**

All iPSC-derived ROs display expression of markers for REC (photoreceptors), SNCG (RGCs, yellow arrows), PROX1 (HCs), AP2α (ACs), PKCα (bipolar cells, white arrows) and CRALBP (Müller glia). REC^+^ photoreceptors are observed on the organoid’s apical edge with SNCG^+^ ganglion cells aligning in the lower central region of the organoid. PKCα^+^ cells are located centrally between the photoreceptor and RGCs, suggesting potential cell-cell connectivity. The interneurons: PROX1^+^ HCs and AP2α^+^ ACs, are found in the putative inner nuclear layer. Müller glial cells are present with their processes spanning the entire retinal structure. This resembles the nascent retina’s correct lamination in both control and patient RO lines. One notable observation is the disruption to interneuron lamination in AC ROs where HCs and ACs are interspersed rather than forming distinct layers.

**
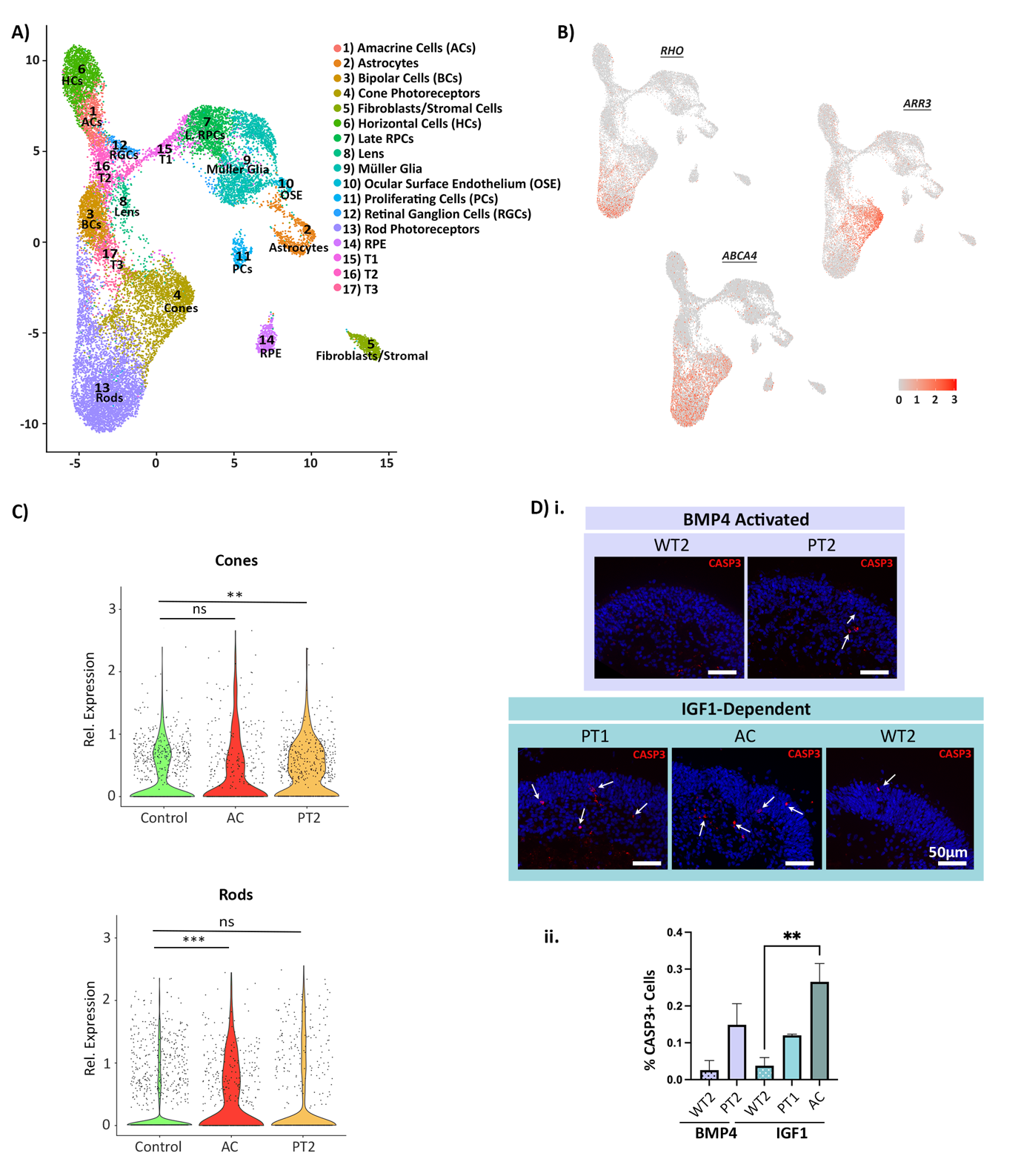
**

**Fig. S4: Single cell RNA-Seq of control and STDG1 ROs.**

1. Integrated UMAP of iPSC-ROs from combined single cells of AC, PT2, WT2 and WT3 ROs at Day 200 of differentiation. 17 distinct cell clusters were identified. Clustering analysis shows the presence of the key major neuronal cell types found in the retina – ACs (C1), Bipolar Cells (C3), Cones (C4), HCs (C6), Müller glia (C9), Retinal Ganglion Cells (C12) and Rods (C13). Extraretinal tissues also feature as clusters including Astrocytes (C2), Fibroblasts/Stromal Cells (C5), Lens (C8), Ocular Surface Epithelium (C10), RPE (C14). Precursor and Progenitor populations are also present with Late RPCs (C7), proliferating cells (PCs) (C11) and the transient progenitor populations T1, T2 and T3 (C15, C16, C17).
2. Individual UMAPs identifying rod and cone clusters by their expression of *RHO* and *ARR3* respectively. *ABCA4* expression is observed in the rod and cone clusters confirming its expression in both of these photoreceptor subtypes.
3. Comparisons of *CASP3* expression on transcript level in cone and rod photoreceptors of STGD1 and WT ROs. In PT2 cones, there is a significant elevation of *CASP3* expression whereas no changes are observed in the AC line. In rods, the opposite is true.
4. (i) On protein level, evidence of activated CASP3 expression is seen in most iPSC-ROs via immunofluorescence. (ii). When quantified, AC displays the highest quantity of CASP3^+^ cells which is statistically significant, followed by PT1 and PT2 (but with no significance) when compared with the WT2 ROs derived in their respective protocols. ** = p-val 0.01.

**
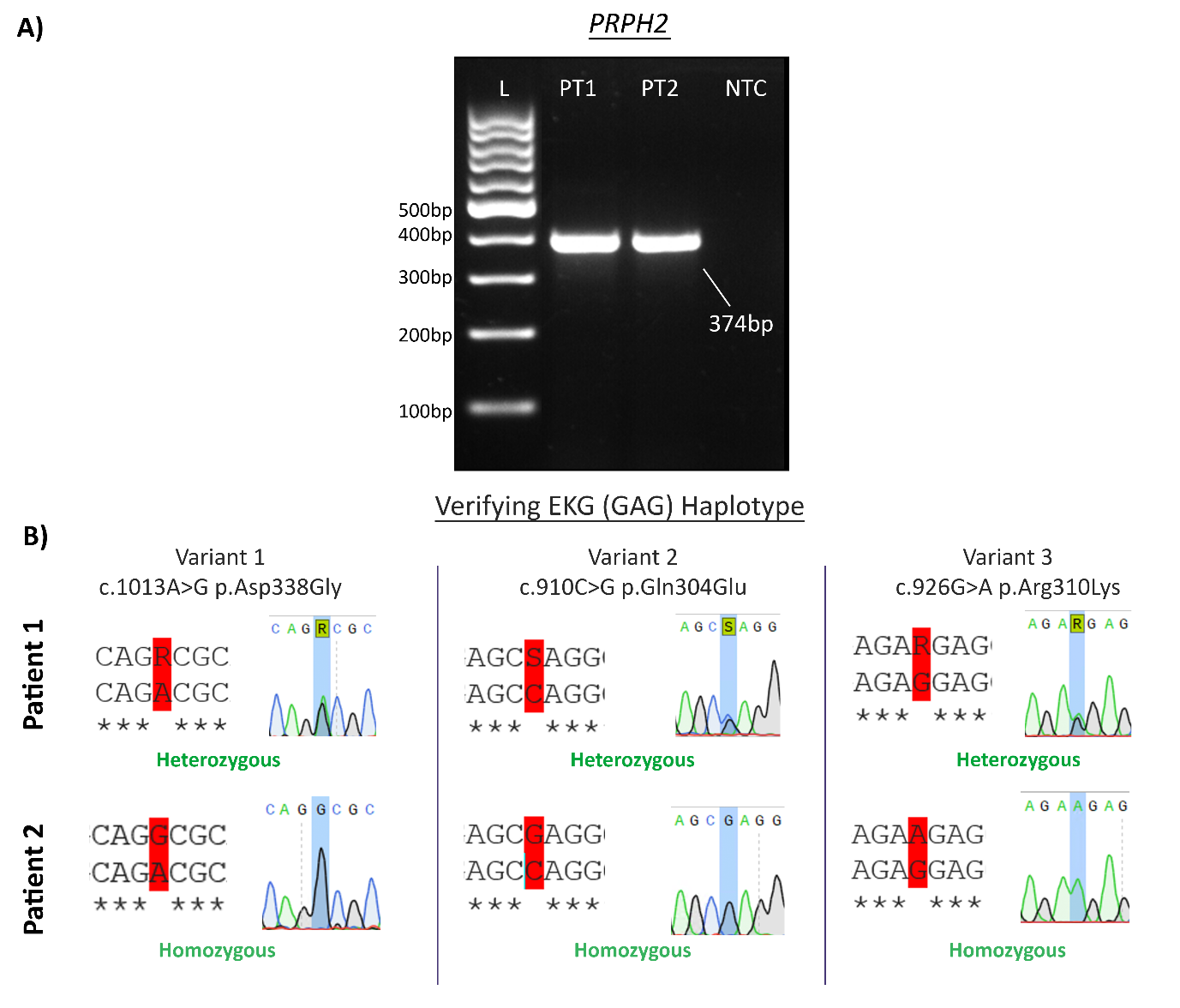
**

**Fig. S5: PT1 and PT2 STGD1 cases carry potential trans-acting modifier haplotype in PRPH2.**

1. Agarose gels displaying PCR amplified products from DNA of both PT1 and PT2 alongside a 100bp ladder (L). Products corresponding to the expected band size for the EKG (GAG) haplotype in *PRPH2* consisting of Variant 1 (c.1013A>G), Variant 2 (c.910C>G) and Variant 3 (c.926G>A) were observed at 374bp. Non-template controls (NTC) were included.
2. Sanger results from the sequenced PCR products. DNA alignments between reference (bottom row) and Sanger sequences (top row) display mismatches, corresponding to the nucleotide peaks in the adjacent Sanger traces. Variants in the EKG haplotype of *PRPH2* were present in heterozygous state in PT1 and homozygous state in PT2.

**
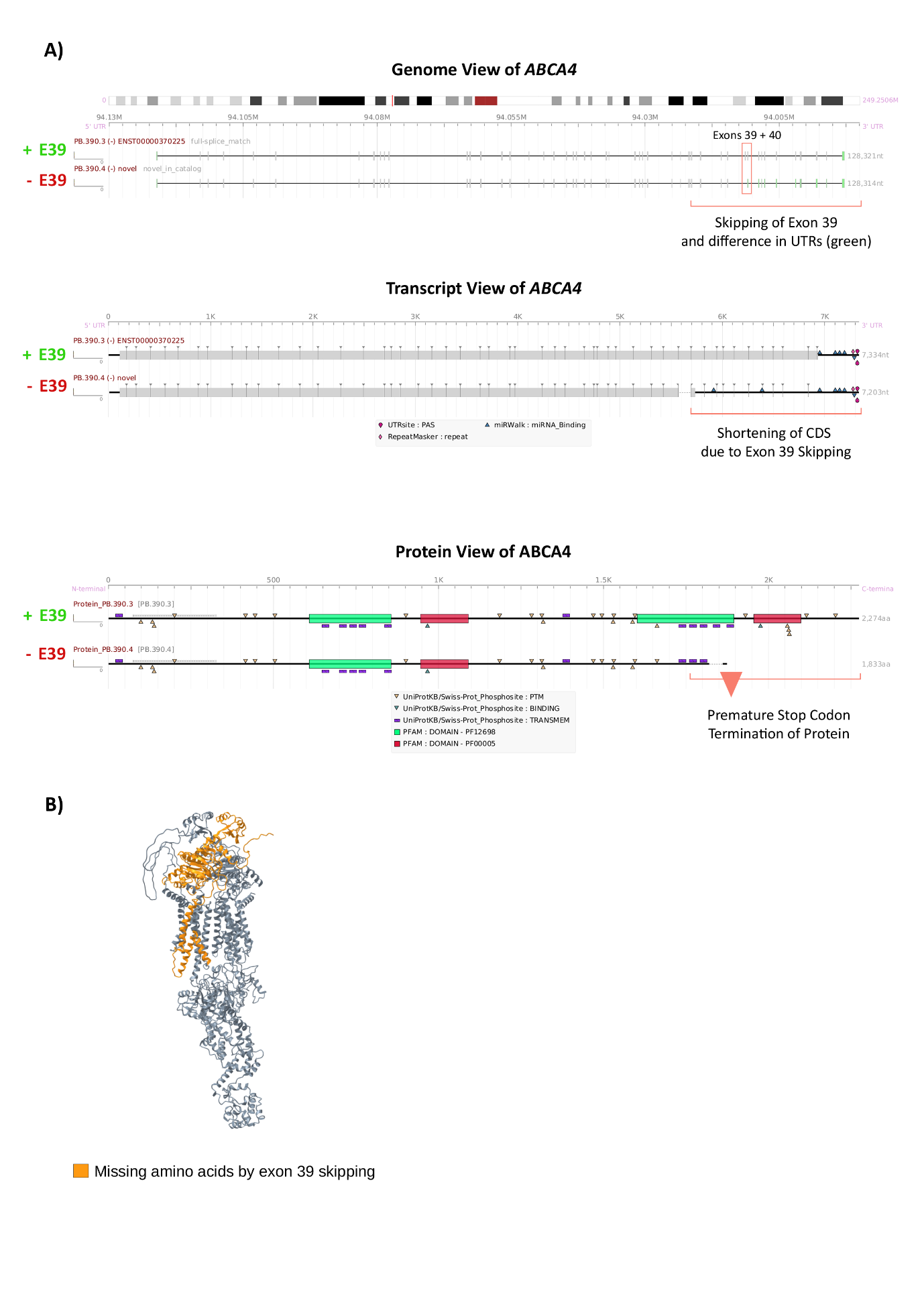
**

**Fig. S6: Visualisation of the functional effects of c.5461-10T>C based on PT1 *ABCA4* PacBio Iso-Seq transcripts**

1. Genomic, transcriptomic and protein view of the effects on *ABCA4* elicited by the c.5461-10T>C variant with/without exon 39 (+E39/-E39) in PT1. Skipping of exon 39 generates a longer 3’ untranslated region (3’ UTR) in PT1 (-E39 (red) in the genome view), and a significant shortening of the coding sequence (CDS) as a result of a premature stop codon (transcript view). This leads to the truncation of two ABCA4 functional domains (protein view).
2. The amino acids affected by variant exon skipping are observed in the 3D structure of ABCA4 protein.
